# Pharmacological inhibition of Lin28 promotes ketogenesis and restores lipid homeostasis in models of non-alcoholic fatty liver disease

**DOI:** 10.1038/s41467-022-35481-1

**Published:** 2022-12-26

**Authors:** Evangelia Lekka, Aleksandra Kokanovic, Simone Mosole, Gianluca Civenni, Sandro Schmidli, Artur Laski, Alice Ghidini, Pavithra Iyer, Christian Berk, Alok Behera, Carlo V. Catapano, Jonathan Hall

**Affiliations:** 1 Department of Chemistry and Applied Biosciences, Institute of Pharmaceutical Sciences, ETH Zurich, Zurich, Switzerland; 2 Tumor Biology and Experimental Therapeutics, Institute of Oncology Research (IOR), Università della Svizzera Italiana (USI), Bellinzona, Switzerland

**Keywords:** Target validation, Metabolic disorders, Metabolic pathways, Non-alcoholic fatty liver disease

## Abstract

Lin28 RNA-binding proteins are stem-cell factors that play key roles in development. Lin28 suppresses the biogenesis of let-7 microRNAs and regulates mRNA translation. Notably, let-7 inhibits Lin28, establishing a double-negative feedback loop. The Lin28/let-7 axis resides at the interface of metabolic reprogramming and oncogenesis and is therefore a potential target for several diseases. In this study, we use compound-C1632, a drug-like Lin28 inhibitor, and show that the Lin28/let-7 axis regulates the balance between ketogenesis and lipogenesis in liver cells. Hence, Lin28 inhibition activates synthesis and secretion of ketone bodies whilst suppressing lipogenesis. This occurs at least partly via let-7-mediated inhibition of nuclear receptor co-repressor 1, which releases ketogenesis gene expression mediated by peroxisome proliferator-activated receptor-alpha. In this way, small-molecule Lin28 inhibition protects against lipid accumulation in multiple cellular and male mouse models of hepatic steatosis. Overall, this study highlights Lin28 inhibitors as candidates for the treatment of hepatic disorders of abnormal lipid deposition.

## Abstract (web summary)

The Lin28/let-7 axis regulates metabolic pathways in normal and pathological contexts. Here the authors show that pharmacological inhibition of Lin28 protects against lipid accumulation in multiple preclinical models of nonalcoholic fatty liver disease.

## Introduction

LIN28 (Lin28A) and its homolog LIN28B (Lin28B) are highly conserved RNA-binding proteins (RBPs) that coordinate fundamental signalling networks in embryonic development, tumorigenesis, tissue regeneration and metabolism^1–4^. During the early stages of development, the expression of Lin28A is generally high, while postnatally it is restricted to selected tissues^5^, such as the hypothalamus, where it controls glucose metabolism^6^. Lin28 proteins bind to conserved sites in the 3’UTRs of a wide array of messenger RNAs, modulating their translation^7,8^, as well as to the terminal loops of let‐7 precursors, where they block processing into mature miRNAs^9–12^. Moreover, Lin28 mRNAs are let-7 targets themselves, establishing a double-negative feedback loop^13,14^.

In transgenic mice overexpressing Lin28A, insulin sensitivity and glucose tolerance are enhanced^15,16^, whereas mice overexpressing let-7 exhibit the reverse phenotype^17^. Lin28A increases the translation of mRNAs for several metabolic enzymes^18^, and the Lin28/let-7 axis regulates the expression of multiple members of the insulin-PI3K-mTOR pathway^15,17,19–23^. In this way, the Lin28/let-7 balance fine-tunes the output of metabolic signalling cascades. Increased expression of Lin28 (and coordinative repression of let-7) is observed in many primary human tumours, and the proteins have been shown to drive malignant transformation in various cancers^24^. Cancer cells maintain high rates of glucose metabolism to meet their bioenergetic and biosynthetic demands (Warburg effect)^25^. Here, let-7 serves to repress important oncogenes and regulators of mitogenic pathways, such as *K-RAS* and *c-MYC*^26,27^, which play key roles in metabolic reprogramming in cancer^28,29^. Thus, the Lin28/let-7 axis sits firmly at the interface of metabolic reprogramming and malignant growth, which is consistent with recent works, for example showing: that the Lin28/let-7 axis facilitates aerobic glycolysis while inhibiting mitochondrial oxidative phosphorylation in hepatocellular carcinoma^30^; that Lin28B enhances glycolysis and lactate secretion to promote cancer stemness^31^; and that Lin28 promotes de novo fatty acid synthesis in liver cancer^32^. Together, these reports highlight the Lin28/let-7 interaction as a potential new drug target, possibly for several diseases associated with misregulation of the PI3K pathway and mTOR signalling.

Small-molecule ligands offer a direct means to identify disease areas in which a new target can be effectively and safely exploited. The search for small-molecule inhibitors of the Lin28/let-7 interaction began shortly after the RNA-protein complex was characterized^33^. Although RNA-protein interactions are commonly considered as challenging molecular targets^34,35^, a few groups have identified small-molecule inhibitors of Lin28^36–42^. However, most of these compounds are insufficiently developed for use in vivo, due to their largely untested pharmacokinetic properties. We identified the small molecule C1632 from an innovative high-throughput screen of 16’000 drug-like compounds^37^. It inhibits Lin28 binding to let-7 precursors in primary cells and mouse embryonic stem cells at micromolar activity and has an excellent selectivity profile according to the PubChem database^37^. Compound C1632 is arguably unique amongst the available small-molecule Lin28 inhibitors because it has proven suitability for use in vivo^43^.

In this study, we demonstrate that Lin28 inhibition by C1632 causes metabolic alterations in mice, manifested by increased ketogenesis and a concomitant suppression of de novo lipogenesis. We find that the Lin28/let-7 axis regulates NCoR1, a co-repressor of peroxisome proliferator-activated receptor-alpha (PPARα), which is the major transcriptional controller of ketogenesis. We confirm that let-7 miRNAs target the 3′UTR of NCoR1 mRNA to decrease NCoR1 protein synthesis. Moreover, our data suggest that Lin28 inhibition leads to repression of insulin/AKT and NCoR1 in the liver, with subsequent induction of the ketogenic pathway by independent and additive pathways. We show that Lin28 inhibition enhances lipid catabolism and ketogenesis, whilst repressing SREBP1-mediated anabolic lipogenesis, which together reduces intracellular lipid accumulation in HepG2 and AML12 cells. Finally, C1632 treatment activates an antisteatotic program in both genetic and dietary mouse models of non-alcoholic fatty liver disease (NAFLD), suggesting that a moderate pharmacological inhibition of Lin28 might be beneficial for the prevention or treatment of NAFLD.

## Results

### Pharmacological inhibition of Lin28/let7 by compound C1632 in liver and skeletal muscle of mice

Low-molecular-weight compounds are valuable tools to probe the functions of proteins in biological mechanisms^44^. Ideally, they are active at low concentrations, they are selective for their target and they have appropriate pharmacokinetic properties for use in vivo. By testing them in models of disease, including in patients, the potential of a putative new target protein in a given disease indication can be clarified with respect to its safety and its potential therapeutic benefits. We hypothesized that testing a small molecule inhibitor of Lin28 in vivo would yield key insights on its potential value as a target for metabolic diseases.

**Fig. 1:**
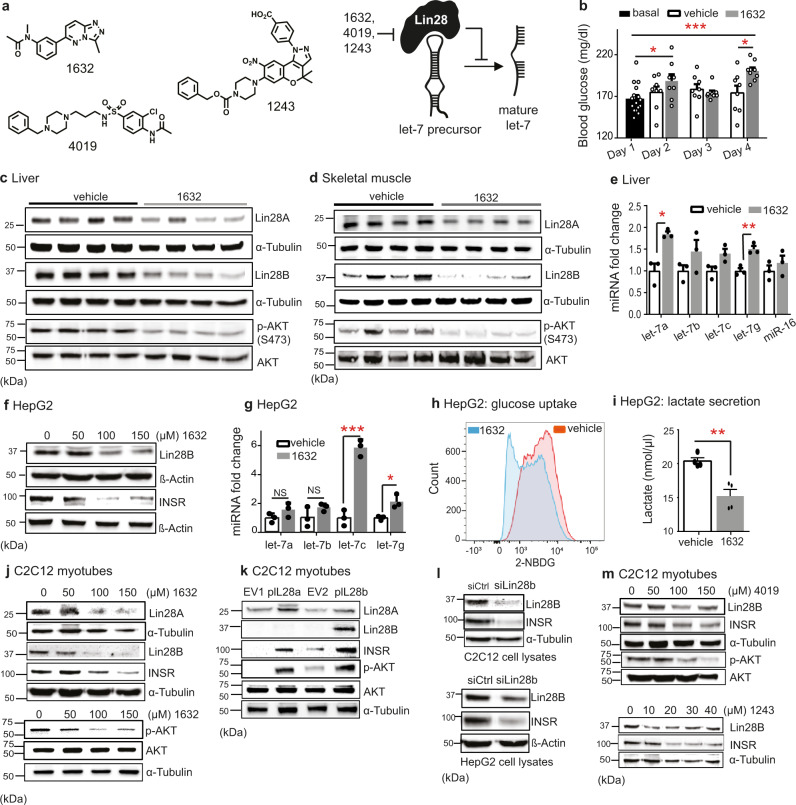
Lin28A/B inhibition suppresses insulin-PI3K-mTOR signalling in liver and skeletal muscle. **a** C1632, C4019 and C1243 inhibit Lin28 and derepress let-7 biogenesis. **b** 7-week-old male C57BL/6J mice received intraperitoneal injections of C1632 (50 mg/kg) or vehicle for 5 consecutive days. Blood glucose was monitored daily (*n* = 8 mice/group; basal vs 1632 Day 2: *P  = 0.0457 and Day 4: ****P < 0.0001; vehicle vs 1632-Day 4: *P  = 0.0224). **c** Immunoblot analysis for Lin28A, Lin28B, p-AKT(S473), AKT in liver lysates in treated mice from (**b**) (Loading control: α-tubulin). **d** Immunoblot analysis in skeletal muscles from (**b**) (Loading control: α-tubulin). **e** Let-7 and miR-16 expression in livers from treated mice (*n* = 3 mice/group; let-7a: *P  = 0.0270; let-7g: **P = 0.0071). **f** Immunoblot analysis for Lin28B and INSR in HepG2 cells after C1632 treatment (Loading control: β-Actin). **g** Let-7 levels in HepG2 cells after C1632 (100 μM) treatment (*n* = 3 biologically independent samples/group; let-7c: ***P = 0.0004; let-7g: *P = 0.0465). **h** Glucose uptake was measured by flow-cytometry in HepG2 cells after C1632 (100 μM) or vehicle treatment, followed by NBDG (representative experiment from four biological replicates). **i** Lactate concentration in medium of HepG2 cells after C1632 (100 μM) or vehicle treatment (*n* = 4 biologically independent samples; **P = 0.0077). **j** Immunoblot analysis for Lin28A, Lin28B and INSR in C2C12 myotubes after C1632 treatment (loading control: α-tubulin). Immunoblot analysis for P-AKT (S473) and AKT in C2C12 myotubes after treatment with C1632 (loading control: α-tubulin). **k** Immunoblot analysis for Lin28A, Lin28B, INSR, p-AKT (S473), AKT, in C2C12 myotubes after transfection with empty vectors (EV), Lin28A/B-overexpression vectors (loading control: α-tubulin). **l** Immunoblot analysis for Lin28B and INSR in C2C12 myotubes after siRNA transfection against Lin28B (20 nM) (loading control: α-tubulin). Immunoblot analysis for Lin28B and INSR in HepG2 cells after transfection with siLin28B (20 nM) (loading control: β-actin). **m** Immunoblot analysis for Lin28B, INSR, p-AKT (S473), AKT in C2C12 myotubes after C4019 treatment. Immunoblot analysis for Lin28B, INSR in C2C12 myotubes after treatment with C1243. Densitometry of immunoblots shown in Fig. S1. Values are mean ± SEM. *P < 0.05; **P < 0.01; ***P  <  0.001; ****P  <  0.0001. Groups were compared using two-tailed unpaired Student’s t-test. Source data provided in Source Data file.

Recently, we developed and used an innovative high-throughput screening assay to identify drug-like repurposing compounds, including two unrelated independent structures (C1632, C4019; Fig. 1a) that inhibit the Lin28/let-7 interaction and restore the processing and function of let-7 miRNAs^37^. Compound C1632 induces differentiation of mouse embryonic stem cells, and inhibits stemness in human cancer cell lines. This compound was originally synthesized in a pharma-industry program as a ligand of interest for the treatment of companion animal anxiety^43,45^. Thanks to its ready availability, it has been tested - and found inactive - in more than 500 in vitro and cellular assays (Pubchem database). Although C1632 is a selective inhibitor of Lin28/let-7, we noted that it also shows an off-target micromolar affinity for BRD4 and CREBBP bromodomains^37^. To date, four reports describe the use of C1632 in rodent models of disease. Albright et al. injected rats with C1632 at 3 and 6 mg/kg and showed protective effects against epileptic seizures^43^. Three independent groups have employed C1632 in models of cancer, to slow tumour growth in breast cancer-derived xenografts at 20 mg/kg or 40 mg/kg^31,46^; and to elicit anti-proliferative effects in oral squamous cell carcinoma xenografts in a combination regime^47^.

To examine the effects of C1632 in vivo, we administered it (50 mg/kg) by intraperitoneal injections on 5 consecutive days to 7-week-old, male C57BL/6J wild-type mice feeding on a normal diet. We observed mildly increased blood glucose levels in treated mice compared with controls (Fig. 1b), consistent with the phenotypes of Lin28A/B knock-out and let-7 transgenic mice, as identified by Daley and colleagues^15,17^. In order to confirm that increased glucose levels were indeed associated with Lin28 inhibition by C1632, we measured levels of Lin28A and Lin28B protein by western blotting from homogenates of liver and skeletal muscle. We have previously shown that Lin28 inhibitors reduce target protein levels in cells^37^, possibly due to the increased expression and activity of let-7 in a feedback mechanism. Lin28A and Lin28B were reduced by approximately 20% and 40%, respectively, in liver (Fig. 1c, Fig. S1a), and by approximately 30% and 60% in skeletal muscle (Fig. 1d, Fig. S1b), consistent with a broad distribution and activity of C1632 in vivo. Furthermore, hepatic let-7a, 7b, 7c and 7g were increased by 1.4-2.0-fold in C1632-treated compared to untreated mice, whereas miR-16 as a negative control was unaffected (Fig. 1e). Lin28 proteins promote the insulin-PI3K-mTOR pathway, which integrates diverse hormonal and nutritional cues in order to regulate metabolic balance^15,48^. Therefore, we examined whether the C1632 treatment of mice inhibited the insulin-PI3K-mTOR pathway. Indeed, AKT Ser473 phosphorylation (p-AKT) was reduced in the liver and skeletal muscle from C1632-treated mice over the control group (Fig. 1c, d, Figs. S1a, 1b), while serum insulin levels remained unchanged (Fig. S2).

In order to support the aforementioned data obtained in vivo, we performed selected experiments in the human HepG2 liver carcinoma cell line, in murine C2C12 skeletal myotubes and in murine AML12 hepatocytes. Consistent with the results obtained from mouse liver samples, treatment of HepG2 cells with C1632 for four days led to reduced levels of Lin28B, p-INSR, INSR proteins (Fig. 1f, Fig. S1c, Fig. S3a) and increases in let-7c and let-7g miRNAs (Fig. 1g). Moreover, glucose uptake into HepG2 cells was decreased by C1632, measured with a 2-[N-(7-nitrobenz-2-oxa-1,3-diazol-4-yl)amino]-2-deoxy-D-glucose (2-NBDG) assay (Fig. 1h), whereas lactate secretion was concomitantly decreased (Fig. 1i). These results were consistent with the reported positive regulation of glucose uptake and glycolytic conversion to lactate by Lin28^30^. Finally, C1632 treatment of C2C12 myotubes decreased levels of Lin28A, Lin28B, p-AKT, p-INSR, and INSR proteins (Fig. 1j, Fig. S1d, Fig. S3b), whereas over-expression of Lin28A or Lin28B in the same cells produced increases in p-AKT and INSR (Fig. 1k, Fig. S1e).

It is prudent to replicate the phenotypic effects obtained with a small-molecule tool compound using complementary ligands^44^. This helps ensure that phenotypic observations of interest do not derive from any off-target effects of the tool compound. This can be accomplished using additional compounds with dissimilar structures or by genetic inhibition of the target. Thus, treatment of C2C12 cells and/or HepG2 cells with Lin28 inhibitors C4019 and C1243 (discovered by us^37^ and Lim et al^38^, respectively), as well as an siRNA targeted to Lin28B^49^, reduced Lin28B, INSR and p-AKT (Fig. 1l, m, Fig. S1f, S1g) and lactate secretion (Fig. S4a, b) similarly to compound C1632.

### Inhibition of Lin28/let-7 causes ketogenesis in mice via let-7-mediated suppression of NCoR1

**Fig. 2:**
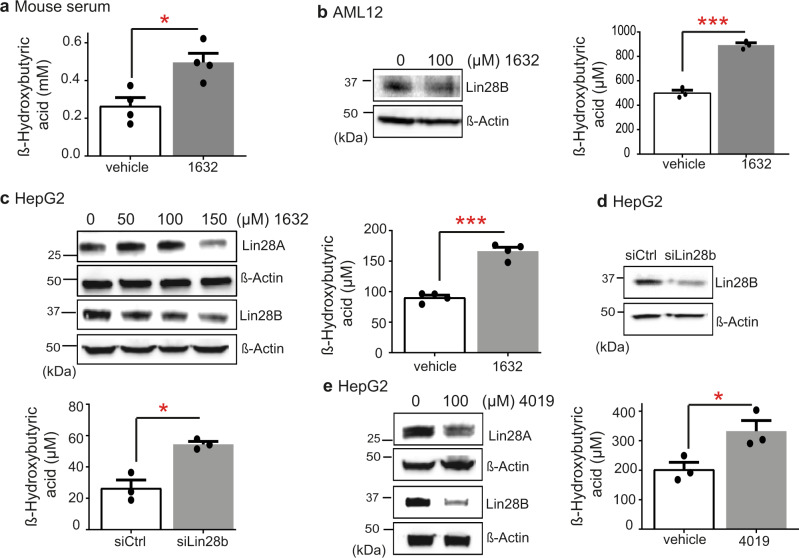
The small molecule Lin28 inhibitor C1632 promotes ketogenesis. **a** 7-week-old male C57BL/6J wild-type mice received intraperitoneal (IP) injections of C1632 (50 mg/kg) or vehicle for 5 consecutive days, while mice were fed *ad libitum*. Serum β-OHB levels were measured in the indicated groups (*n* = 4 mice/group, *P = 0.0129). **b** AML12 cells were treated with 0 or 100 μΜ of compound C1632 in the presence of a fatty acid (F.A.) mixture of oleate and palmitate for 72 h. β-OHB levels were measured in the growth medium of AML12 cells and β-OHB levels were normalized to total protein of the corresponding samples (*n* = 3 biologically independent samples/group, ***P = 0.0003). **c** HepG2 cells were treated for 4 days with C1632 in the presence of a F.A. mixture of oleate and palmitate. Lin28A/Lin28B inhibition was examined by immunoblot analysis. β-OHB levels were measured in the growth medium of cells treated with vehicle or C1632 (100 μM) and β-OHB levels were normalized to total protein of the corresponding samples (*n* = 4 biologically independent samples/group, ***P = 0.0002). **d** HepG2 cells were transfected with siLin28b. 48 h after the transfection the medium was changed, and cells were incubated with medium containing F.A. for 24 h. Lin28B inhibition was examined by immunoblot analysis. β-OHB levels were measured in the growth medium and β-OHB levels were normalized to total protein of the corresponding samples (*n* = 3 biologically independent samples/group, *P = 0.0227). **e** HepG2 cells were treated for 3 days with C4019 (100 μM) in the presence of F.A. Lin28A/Lin28B inhibition was examined by immunoblot analysis. β-OHB levels were measured in the growth medium and β-OHB levels were normalized to total protein of the corresponding samples (*n* = 3 biologically independent samples/group, *P = 0.0469). Values are mean ± SEM. *P < 0.05; **P < 0.01, ***P  <  0.001. Groups were compared using two-tailed unpaired Student’s t-test. Source data are provided as a Source Data file.

The treatment of 7-week-old, male C57BL/6J wild-type mice with regular injections of compound C1632 did not produce any obvious adverse effects nor alter the body weight of the mice (Fig. S5a–c). However, in preliminary experiments, some mice exuded a mild odour of acetone after 5 days, which is associated with ketogenesis. Ketogenesis is the synthesis of ketone bodies by β-oxidation of fatty acids in hepatic mitochondria. Ketone bodies are catabolized to support the metabolic requirements of vital organs when glucose availability is low^50,51^. Increased ketogenesis is linked to impaired signalling in the insulin-PI3K-mTORC1 pathway^52^, and is observed after treatment with rapamycin, an allosteric inhibitor of mTORC1^52,53^. Thus, our indications of ketogenesis by C1632 treatment were consistent with the suppression of insulin-AKT signalling in the liver. Indeed, serum levels of β-hydroxybutyrate (β-OHB), the most abundant ketone body^54^, were significantly higher in C1632-treated mice compared to controls (Fig. 2a). We observed similar increases in serum β-OHB levels in a different strain of 7-week-old, male mice (Crl:NU(NCr)-Foxn1nu) treated for 5 days or 3 weeks (Fig. S6a, S6b) and within few hours after administration of compound C1632 (Fig. S6c). The C1632-induced rise in β-OHB levels was on a comparable scale to that stimulated by a regimen of reduced food intake^55^, although changes in β-OHB were milder than those triggered by fasting^56^ or diabetes^57^. Furthermore, glucose tolerance was only moderately affected in C57BL/6J mice (Fig. S7a), while blood glucose levels, glucose and insulin tolerance were unaffected in the nude mice upon C1632 treatment (Fig. S7b–f), suggesting that the main effect of Lin28 inhibition was on lipid metabolism. Consistent with these observations in vivo, treatment of murine AML12 hepatocytes and human HepG2 cells in the presence of added fatty acids with C1632, as well as C4019 and siLin28B, increased secretion of β-OHB by approximately two-fold (Fig. 2b–e). Taken together, these data indicated that inhibition of Lin28A/B activates ketogenesis in a hepatic cell-autonomous fashion.

**Fig. 3:**
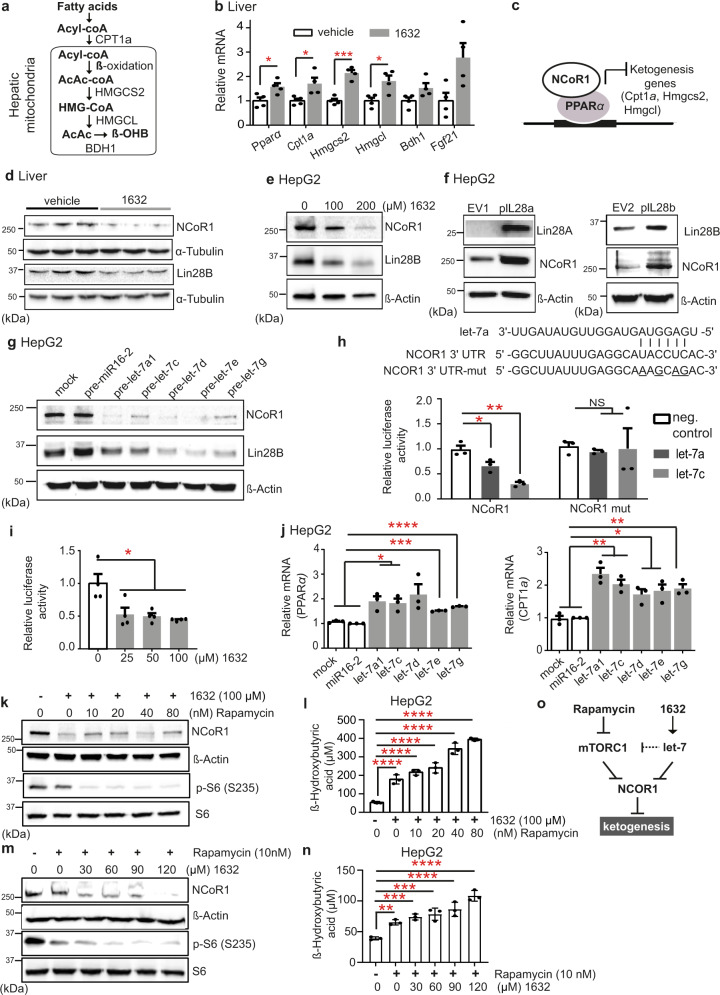
Lin28/let-7 regulates the ketogenesis repressor NCoR1. **a** Ketogenesis pathway. **b** Expression of ketogenic genes from C1632-treated 7-week-old, male, wild-type mice after 5 daily IP injections (50 mg/kg) (*n* = 4 mice/group; Pparα: *P  = 0.0285; Cpt1a: *P  = 0.0338; Hmgcs2: ***P = 0.0006; Hmgcl: *P  = 0.0272). **c** NCoR1 represses PPARα-controlled ketogenesis. **d** Immunoblot analysis for NCoR1 and Lin28B in liver tissues from treated mice as described in (**b**). **e** Immunoblot analysis for NCoR1 in HepG2 cells after 1632-treatment. **f** Analysis for NCoR1 in HepG2 cells with/without Lin28A/B overexpression. **g** Immunoblot analysis for NCoR1 and Lin28B in HepG2 cells after pre-let-7 transfections (pre-miR-16-2 as control). **h** Predicted base-pairing between let-7 and NCoR1 3’UTR. Wild-type or mutated (mut) NCoR1 sequences inserted into dual-luciferase vectors for assays in HEK293T cells (*n* = 3 biologically independent samples/group; let-7a: *P  = 0.0447; let-7c: **P  = 0.0064). **i** HEK293T cells were treated with C1632 and wild-type plasmid and luciferase activity was assayed (*n* = 4 independent samples; 0 vs 25 μM: *P  = 0,0400; 0 vs 50 μΜ: *P  = 0,0340; 0 vs 100 μM: *P  = 0,0299). **j** Relative PPARα and CPT1a mRNA levels in HepG2 cells transfected with pre-let-7 (*n* = 3 independent samples/group; (Ppara) mock vs let-7a1: *P  = 0.0171; mock vs let-7c: *P  = 0.0157; mock vs let-7e: ***P  = 0.0002; mock vs. let-7g: ****P < 0.0001; (Cpt1a) mock vs let-7a1: **P  = 0.0074; mock vs let-7c: **P  = 0.0052; mock vs let-7d: *P  = 0.0231; mock vs let-7e: *P  = 0.0300; mock vs let-7g: **P  = 0.0065). **k, m** HepG2 cells treated with C1632/rapamycin combinations. Rapamycin activity was validated by measuring effects on P-S6. NCoR1 levels were determined by immunoblot analysis. **l, n** β-OHB levels in the medium of cells treated as indicated (*n* = 3 independent samples/group; ****P < 0.0001; vehicle vs. Rapamycin (10 nM): **P = 0.0056; vehicle vs. Rapamycin (10 nM) +1632 (30 μM): ***P = 0.0006; vehicle vs. Rapamycin (10 nM)+1632 (60 μM): ***P = 0.0002; vehicle vs. Rapamycin (10 nM)+1632 (90 μM): ****P < 0.0001; vehicle vs. Rapamycin (10 nM)+1632 (120 μM): ****P < 0.0001). **o** Schematic showing effects of Rapamycin and C1632: Rapamycin inhibits mTORC1 and prevents nuclear translocation of NCoR1^52^, whereas let-7 inhibits NCoR1 synthesis (this work). Densitometry of immunoblots are provided in Fig. S9a-f. Values are mean ± SEM. *P < 0.05; **P < 0.01; ***P  <  0.001, ****P  <  0.0001. Groups were compared using two-tailed unpaired Student’s t-test and 1-way ANOVA with Bonferroni’s post hoc test. Source data provided in Source Data file.

Next, we examined the expression of known regulators of ketogenesis in livers of vehicle- and C1632-treated mice. mTORC1 signalling suppresses ketogenesis^52,58–60^ by repressing peroxisome proliferator-activated receptor alpha (PPARα), the main transcriptional activator of ketogenesis in liver^61^. This occurs through recruitment of the nuclear receptor co-repressor 1 (NCoR1) at PPARα response element (PPRE)-containing promoters^52^, activated by AKT^58^ and S6 kinase 2 (S6K2)^60^. β-OHB is produced in a series of enzymatic steps from fatty acids in a well-characterized pathway that is regulated by PPARα (Fig. 3a). Hence, we examined the mRNA levels of PPARα and its downstream gene targets from the pathway. We found that 3-hydroxy-3-methylglutaryl (HMG)-CoA synthase 2 (Hmgcs2), 3-hydroxy-3-methylglutaryl-CoA lyase (Hmgcl), 3-hydroxybutyrate dehydrogenase 1 (Bdh1), carnitine palmitoyltransferase 1a (Cpt1a) and fibroblast growth factor 21 (Fgf21) were all up-regulated 1.5-3-fold in the livers of C1632-treated mice compared to controls (Fig. 3b; Fig. S8a, S8c). Taken together, these findings demonstrated that small-molecule pharmacological inhibition of Lin28 activates the ketogenic pathway in chow-fed mice, leading to elevated production of β-OHB. Although high circulating levels of β-OHB are associated with severe insulin deficiency and diabetic ketoacidosis^62^, mild ketosis induced by ketogenic diets or β-OHB supplementation confers positive effects in several diseases, including cancer, neurodegenerative diseases, heart failure, polycystic kidney disease and fatty liver disease^50,51,54,55,63–66^.

PPARα is the major transcription factor controlling fatty acid oxidation and ketogenesis^67,68^. In liver, NCoR1 inhibits PPARα activation and its downstream targets, attenuating ketogenesis (Fig. 3c)^52,58,60,69^. The activity of NCoR1 is regulated by insulin-induced AKT phosphorylation at Serine 1460^58^, which is reduced during ketogenesis. Interestingly, NCoR1 protein levels decrease in ketogenic conditions^69,70^. Consistent with this role, we found that levels of the parent NCoR1 protein in the livers of C1632-treated 8-week-old male wild-type mice were reduced by approximately two-fold (Fig. 3d, Fig. S9a). Furthermore, C1632 treatment lowered levels of NCoR1 in HepG2 cells (Fig. 3e, Fig. S9b), whereas the concomitant overexpression of NCoR1 eliminated the effect of C1632 on β-OHB secretion and attenuated the induction of PPARα target genes *Mcad* and *Cpt1a*. This result confirmed that loss of NCoR1 activity was required for the C1632 ketogenic effect (Fig. S10a, 10b).

Although the mTORC1-dependent regulation of ketogenesis is already described, the control of NCoR1 expression and function by the Lin28-let7 axis has not been reported. Hence, we transfected plasmids expressing Lin28A, Lin28B or empty vectors (EV) into HepG2 cells and assayed levels of NCoR1 protein. Accordingly, both vectors yielded an increase in NCoR1 compared to controls (Fig. 3f, Fig. S9c). The 3’UTR of NCoR1 is predicted by Targetscan - the gold-standard miRNA-targeting software^71^ - to be inhibited by let-7 miRNAs. To help ascertain whether induction of PPARα by compound C1632 occurred at least partly via increased activity of let-7, we transfected HepG2 cells with precursors of let-7a1, let-7c, let-7d, let-7e and let-7g, and pre-miR-16-2 (non-targeting control) and extracted RNA and protein from treated cells (Fig. S9d, Fig. S11a-d). As expected, let-7 miRNAs strongly repressed Lin28B protein^10^ (Fig. 3g), whereas expression of NCoR1 was even more strongly decreased (Fig. 3g, Fig. S9d), with modest changes in NCoR1 mRNA (Fig. S12). We cloned the putative let-7 binding site from the NCoR1 3’UTR into a luciferase psiCHECK-2 reporter vector; a binding site sequence containing four mutations served as a negative control. Upon co-transfection of the vectors with let-7a and let-7c into HEK293 cells, we observed repression of luciferase activity from the predicted let-7 recognition site compared to that of the negative control (Fig. 3h). Accordingly, treatment of HEK293T cells expressing the reporter gene with C1632 also decreased Lin28B protein level (Fig. S13) and luciferase activity (Fig. 3i), confirming that the small molecule regulates NCoR1 via let-7 repression of its 3’UTR. Given the role of NCoR1 on the expression of ketogenic genes^58,69^, and its regulation by let-7, we investigated the effects of exogenous let-7 expression on PPARα in HepG2 cells. In line with the fact that PPARα positively regulates its own expression^72^, transfection of let-7 precursors into HepG2 cells raised levels of PPARα mRNA and protein (Fig. 3j, Fig. S14), as well as that of CPT1a, a key enzyme in liver ketogenesis (Fig. 3j). Collectively, these data indicate that the expression of NCoR1 is regulated by the Lin28/let-7 axis, inhibition of which lowers NCoR1 activity, thereby restoring PPARα-mediated expression of enzymes in the ketogenesis pathway.

To provide additional insight on the possible individual contributions of mTOR inhibition and let-7 activity to the overall effects of C1632 on NCoR1 function and the ketogenic program in human cells, we performed combination treatments using rapamycin and C1632 in cells (Fig. 3k–o, Figs. S9e, S9f). The allosteric inhibitor of mTORC1, rapamycin, has been shown previously to elevate ketone bodies by promoting the translocation of NCoR1 to the cytoplasm, leading to the de-repression of downstream PPARα targets^52^. In one scheme, we treated cells with increasing concentrations of rapamycin doses (0, 10, 20, 40, 80 nM) in the presence of a constant dose of C1632 (100 μM). In the absence of rapamycin, C1632 at 100 μM raised β-OHB by approximately three-fold (as before). However, a combination of C1632 with 10-80 nM rapamycin raised β-OHB further, in a concentration-dependent manner, to approximately eight-fold basal levels. In a reverse experiment, a 10 nM treatment of cells with rapamycin increased basal levels of β-OHB by approximately 20%, while its combination with graded concentrations of C1632 (30-120 mM) increased β-OHB further. Notably, levels of NCOR1 protein were largely unaffected by rapamycin addition (Figs. S9e, S9f), consistent with previous reports showing that mTORC1 does not affect NCoR1 protein levels but rather controls its nuclear localization^52,60^. Taken together, the experiments showed that rapamycin and C1632 can function additively via two at least partially independent pathways to release ketogenesis and increase β-OHB levels.

### Pharmacological inhibition of Lin28/let-7 restores lipid homeostasis in murine AML12 and human HepG2 cells

**Fig. 4:**
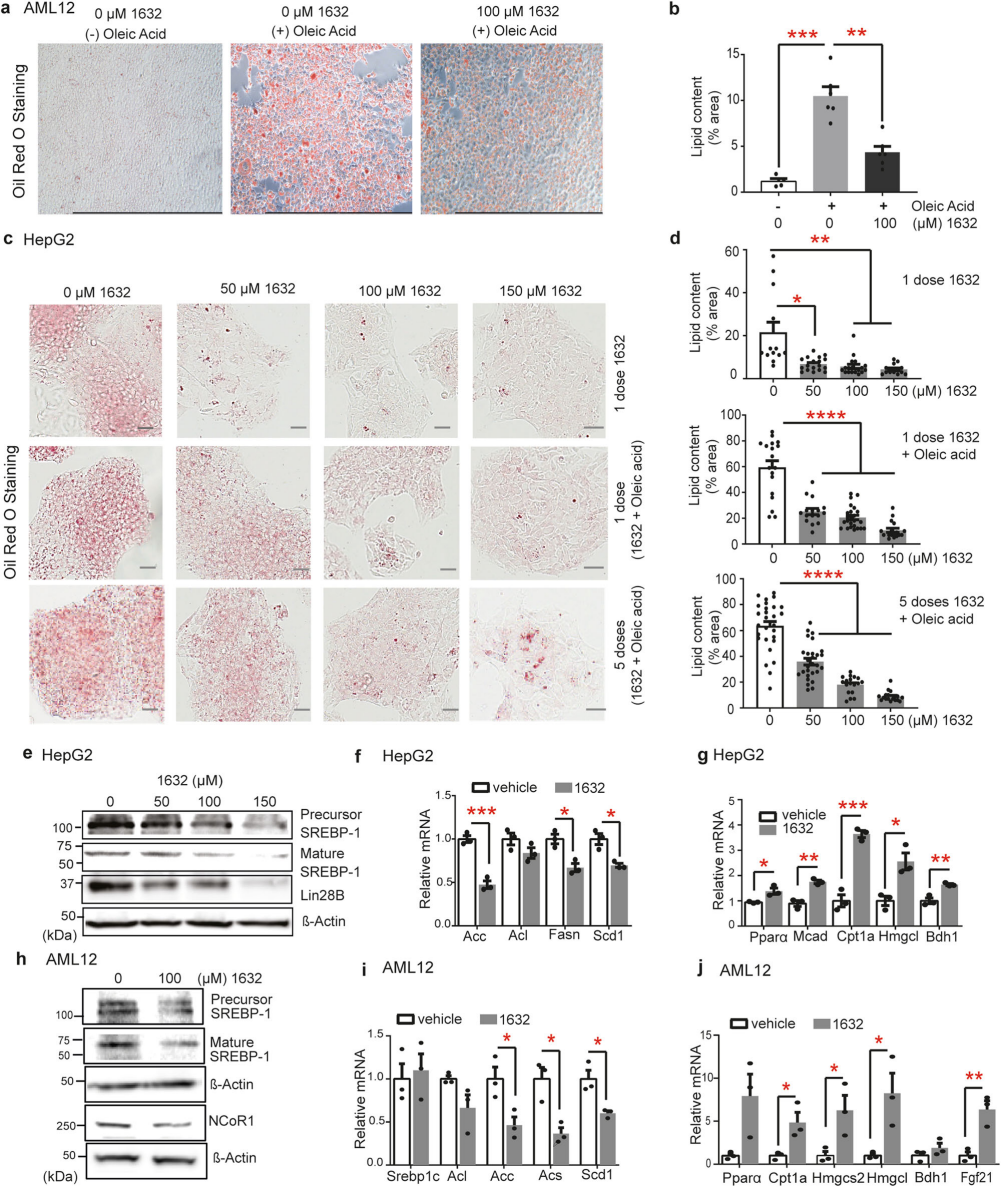
C1632 improves lipid metabolism in hepatocytes in vitro. **a** Representative images of lipids by Oil Red-O staining in AML12 cells treated with C1632, with/without oleic acid for 72 h. Scale bars correspond to 1 mm. **b** Quantification of lipids from Oil Red O-stained AML12 cells (*n* = 4-6 histological images/group; -Oleic Acid/0 μM 1632 vs +Oleic Acid/0 μM 1632, ***P = 0.0001; +Oleic Acid/0 μM 1632 vs +Oleic Acid/100 μM 1632; ***P = 0.0008). Percentage of total histological area is shown. **c** Representative images of lipids by Oil Red-O staining in HepG2 cells treated with increasing concentrations of C1632, with or without oleic acid. Scale bars correspond to 100 μm. **d** Quantification of lipids from Oil Red O-stained HepG2 cells. Percentage total histological area is shown (*n* = 13-24 histological images/group; (1 dose 1632), 0 vs 50 μΜ, *P = 0.0105; 0 vs 100 μΜ, **P = 0.0070; 0 vs 150 μΜ, **P = 0.0040; (1 dose 1632 + Oleic Acid), ****P < 0.0001; (5 doses 1632 + Oleic Acid), ****P < 0.0001). **e** Representative immunoblot analysis for SREBP-1 and Lin28B in C1632-treated HepG2 cells, as described in (**c**). **f, g** Relative mRNA levels of lipogenesis and fatty acid β-oxidation/ketogenesis-related genes in HepG2 cells treated with C1632 (100 μΜ) as described in (**c**), determined using real-time PCR. β-Actin was used as a housekeeping gene (*n* = 3 biologically independent samples/group; Acc, ***P = 0.0009; Fasn, *P = 0.0108; Scd1, *P = 0.0292; Pparα, *P = 0.0222; Mcad, **P = 0.0029; Cpt1a, ***P = 0.0007; Hmgcl, *P = 0.0153, Bdh1, **P = 0.0062). **h** Representative immunoblots showing SREBP-1 and NCoR1 in C1632-treated AML12 cells as described in (**a**). β-Actin was used as a loading control. **i, j** Relative mRNA levels of lipogenesis and fatty acid β-oxidation/ketogenesis-related genes in C1632-treated (100 μΜ) AML12 cells as described in (**a**), as determined using real-time PCR. β-Actin was used as a housekeeping gene (*n* = 3 biologically independent samples/group; Acc, *P = 0.0332; Acs, *P = 0.0134; Scd1, *P = 0.0196; Cpt1a, *P = 0.0326; Hmgcs2, *P = 0.0435; Hmgcl, *P = 0.0362; Fgf21, **P = 0.0084). Densitometric analyses for (**e**) and (**h**) are shown in Fig. S15a, b. Numbers to the left of the blots indicate molecular weight markers. Values are mean ± SEM. *P < 0.05; **P < 0.01; ***P  <  0.001, ****P < 0.0001. Groups were compared using two-tailed unpaired Student’s t-test. Source data provided in Source Data file.

The balance between lipogenesis and fatty acid catabolism maintains lipid homeostasis at the cellular and organismal levels^73^. Disruption of this balance leads to pathological conditions such as non-alcoholic fatty liver (NAFL) and steatohepatitis (NASH), which are characterized by pathological accumulation of lipids in the liver. Moreover, a deficiency in ketogenesis contributes to the development of hepatic steatosis^66,74^. Dietary interventions that enhance ketogenesis can alleviate NAFLD^75^, although excessive ketogenesis can be harmful^76^. A recent study by Zhang and colleagues has shown that Lin28 enhances de novo fatty acid synthesis via translational control of the transcription factor sterol regulatory element-binding protein 1 (SREBP1) and the SREBP cleavage‐activating protein (SCAP)^32^. Our findings suggested an equally important role for Lin28 in ketogenesis. Thus, it seemed plausible that a carefully controlled pharmacological inhibition of Lin28 might yield a dual effect as both an enhancer of ketogenesis and a suppressor of lipogenesis, thereby offering a novel and potentially therapeutic means to restore lipid imbalances in liver disorders. Hence, we first investigated whether compound C1632 could control lipid homeostasis and block oleic acid-induced lipid accumulation in two commonly used cellular models of hepatic steatosis in murine AML12 hepatocytes and human HepG2 cells. Exposure of the non-tumorigenic mouse hepatocyte cells AML12 to oleic acid promoted intracellular lipid accumulation, which was alleviated by C1632 treatment, as shown by Oil Red O staining (Fig. 4a, b). Similarly, in basal conditions, HepG2 cells show low levels of lipid deposits (Fig. 4c, d). Loading cells with oleic acid increased accumulation of lipid droplets. Notably, single and repeated treatments with C1632 exerted a concentration-dependent lipid-lowering effect in both basal and oleic acid-loaded HepG2 cells (Fig. 4c, d). In both these cell types, C1632 treatment lowered SREBP-1 precursor and mature protein levels (together with NCoR1), in agreement with the reported Lin28-regulation of SREBP-1^32^ (Fig. 4e, h, Figs. S15a, 15b). Consistent with these results, the expression of enzymes involved in fatty acid and triglyceride synthesis were decreased (Fig. 4f, i), whereas those involved in mitochondrial β-oxidation and ketogenesis increased (Fig. 4g, j).

### Treatment with the Lin28 inhibitor C1632 prevents the development of NAFLD in Pten^-/-^ mice

To investigate whether promoting ketogenesis and fatty acid catabolism via pharmacological inhibition of Lin28/let-7 could be beneficial in liver disease, we turned to established mouse models of non-alcoholic fatty liver disease (NAFLD). There exist numerous models of NAFLD. Indeed, Friedman *et al* list eleven different mouse models of NAFLD in their recent review, each with its strengths and weaknesses^77^. Mice with liver-specific knockout of Pten (*AlbCrePten*^*flox/flox*^; Pten^-/-^) reproduce many of the features of human NAFLD, including hepatomegaly and steatohepatitis^78,79^. Hepatic deletion of Pten results in overactive AKT, and thereby increases triglyceride (TG) synthesis and causes hepatic steatosis via insulin-induced lipogenesis^79–81^. In the later stages of life (>40 weeks), Pten^-/-^ mice develop adenomas and hepatocellular carcinomas. Thirty-two week-old male Pten^-/-^ mice have enlarged and pale-coloured livers compared to control *Pten*^*flox/flox*^ mice (Fig. S16a–c). Furthermore, livers of Pten^-/-^ mice present characteristic histopathological features of NAFLD, including loss of normal hepatocyte morphology and areas of micro- and macro-steatosis. Enhanced staining for Perilipin2, a main protein contributing to lipid droplet formation, confirmed the previously reported accumulation of large lipid inclusions in the Pten^-/-^ mice (Fig. S16d)^78,79^. Along with Pten depletion, the mice exhibit activation of PI3K-AKT signalling with increased phosphorylation of AKT and high levels of INSR (Fig. S16e). Although western blotting of Lin28A and Lin28B proteins showed similar levels to those of wild-type mice (Fig. S16e), we cannot rule out that Lin28 activity might be regulated post-translationally, for example by the Insulin Receptor-activated Extracellular Signal-regulated Kinases (ERKs), that consistent with previous reports, were found up-regulated in Pten^-/-^ livers^82,83^ (Fig. S16e) and have been shown to phosphorylate Lin28^84,85^. In line with previous studies^78,79,86^, we measured increased hepatic expression of lipogenesis genes (Acs, Acc, Acl, Scd1) (Fig. S16f) and elevated serum levels of the liver damage marker alanine aminotransferase (ALT) (Fig. S16g) in Pten^-/-^ mice compared with controls. Most notably, we also found that the expression of ketogenesis genes (Fig. S16h) and serum levels of β-OHB levels (Fig. S16i) were lower in Pten^-/-^ mice compared with their controls indicating a complex disruption of lipid metabolism in this model of hepatic steatosis.

To determine whether pharmacological Lin28 inhibition could retard the development of liver steatosis, we initially treated male Pten^-/-^ mice at early age (6 weeks old) with C1632 or vehicle for 4 weeks. This short treatment did not lead to changes in liver weight, nor alter histopathological features (Figs. S17a–d). However, it reduced Lin28A and INSR protein levels without changes in serum insulin and free fatty acids (FFA) (Fig. S17e–g), raised serum β-OHB levels (Fig. S17h), and increased the expression of ketogenesis genes (Fig. S17i). Conversely, expression of lipogenesis genes was repressed by C1632 treatment (Fig. S17j), and levels of ALT were lowered (Fig. S17k). Since NASH is characterized by hepatic inflammation, liver injury, and fibrosis, we measured the levels of pro-inflammatory and pro-fibrotic genes as early markers of pathological alterations associated to steatohepatitis in mouse liver. We found that expression of these marker genes was higher in Pten^-/-^ mice compared with controls (Fig. S18a), and that administration of C1632 decreased their values (Fig. S18b).

**Fig. 5:**
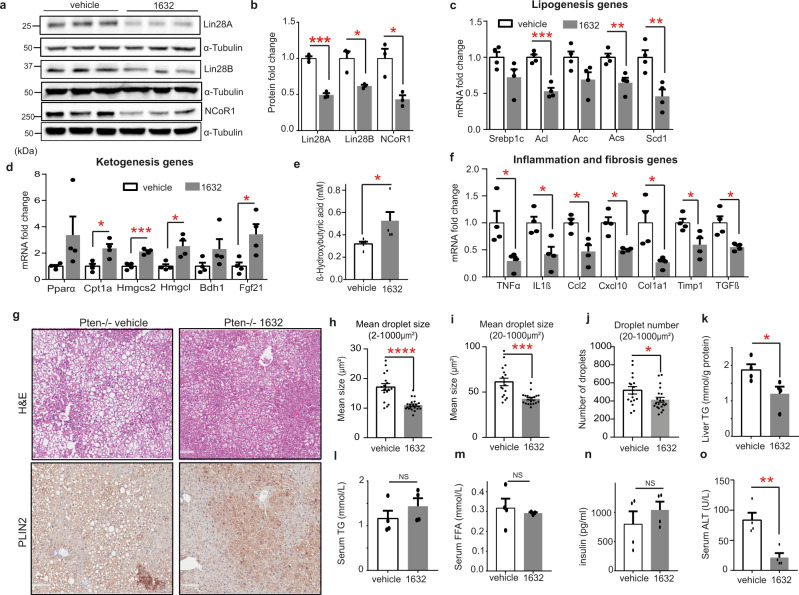
C1632 attenuates hepatosteatosis in a genetic mouse model of NAFLD. **a, b** Representative immunoblot analysis and quantitative densitometric analyses for Lin28A, Lin28B and NCoR1 in livers from 28-week-old Pten^-/-^ male mice treated with vehicle or C1632 (50 mg/kg) after 6 weeks of treatment (3 IP injections per week). α-Tubulin was used as a loading control *(n* = 3 mice/group; Lin28A ***P = 0.0002; Lin28B, *P = 0.0183; NCoR1, *P = 0.0172). **c, d** Relative mRNA levels of lipogenic and ketogenic genes in livers from Pten^-/-^ male mice treated with vehicle or C1632 as described in (**a**) (*n* = 4 mice/group; Acl, ***P = 0.0003; Acs, **P = 0.0076; Scd1, **P = 0.0074; Cpt1a, *P = 0.0181; Hmgcs2, ***P = 0.0005; Hmgcl, *P = 0.0280; Fgf21, *P = 0.0450). β-Actin was used as a housekeeping gene. **e** Serum β-OHB levels in Pten^-/-^mice treated with vehicle or C1632 as described in (**a**) (*n* = 4 mice/group, *P = 0.0331). **f** Relative mRNA levels of inflammation and fibrosis markers in livers from Pten^-/-^ male mice treated with vehicle or C1632 as described in (**a**) (*n* = 4 mice/group; TNFα, *P = 0.0424; IL1β, *P = 0.0183; Ccl2, *P = 0.0119; Cxcl10, *P = 0.0157; Col1a1, *P = 0.0409; Timp1 *P = 0.0356, TGFb, *P = 0.0298). β-Actin was used as a housekeeping gene. **g** Representative Hematoxylin and Eosin (H&E) staining and Perilipin (PLIN2) staining of liver sections from vehicle and C1632 (50 mg/kg)-treated 28-week old Pten^-/-^ mice after 6 weeks of treatment (3 IP injections per week). Liver tissues were collected 2 h after the last C1632 injection. Scale bars correspond to 300 μm. **h–j** Lipid droplet size and number were quantified by Image J (*n* = 17-21 histological images/group, ****P < 0.0001 (**h**), ***P = 0.0004 **(i**), *P = 0.0407 (**j**))**. k** Hepatic triglyceride (TG) content in Pten^-/-^ mice treated with vehicle or C1632, as described in **(a)** (*n* = 4 mice/group, *P = 0.0412). **l-o** Serum TG (*n* = 4 mice/group), free fatty acids (FFA) (*n* = 3-4 mice/group,), insulin (*n* = 4 mice/group), and ALT levels (*n* = 4 mice/group, **P = 0.0077) in Pten^-/-^ mice treated with vehicle or C1632, as described in **(a)**. Numbers to the left of the blots indicate molecular weight markers. Values are mean ± SEM. *P < 0.05; **P < 0.01; ***P  <  0.001, ****P < 0.0001. Groups were compared using two-tailed unpaired Student’s t-test. Source data are provided as a Source Data file.

These encouraging outcomes confirmed that in Pten-deficient mice, pharmacological Lin28 inhibition promoted ketogenesis in spite of the constitutive PI3K-AKT-mTOR signalling. Therefore, we repeated the C1632 treatment in 22 weeks-old Pten^-/-^ male mice with three injections of C1632 per week for six weeks. This longer experiment also did not affect the total liver or bodyweight of the mice (Fig. S19a–c). As expected, levels of Lin28A/B and NCoR1 proteins were decreased (Fig. 5a, b), and similar changes were observed for lipogenesis- and ketogenesis-related mRNAs (Fig. 5c, d), as well as serum β-OHB levels (Fig. 5e) as in the short-term experiments. Moreover, C1632 treatment decreased the hepatic expression of inflammatory and fibrotic genes (Fig. 5f)

Importantly, histological analysis of livers by hematoxylin and eosin (H&E) staining, as well as perilipin2 staining, from C1632-treated mice (Fig. 5g) now revealed an overall decline in the mean size of lipid droplets (Fig. 5h) and a decrease in the number and size of large lipid droplets (Fig. 5i, j). This was consistent with reduced macrovesicular steatosis in the hepatocytes. Accordingly, hepatic triglyceride levels were also reduced, independent of serum TG, FFA and insulin levels (Fig. 5k–n). In line with the histological improvements, C1632-treated Pten^-/-^ mice showed lower serum ALT levels (Fig. 5o). Taken together, this data suggested that small molecule inhibition of Lin28 with for example C1632 has the potential to shift lipid homeostasis in liver towards a healthier state in a genetic mouse model of NAFLD.

### Pharmacological inhibition of Lin28 improves diet-induced NAFLD in mice

**Fig. 6:**
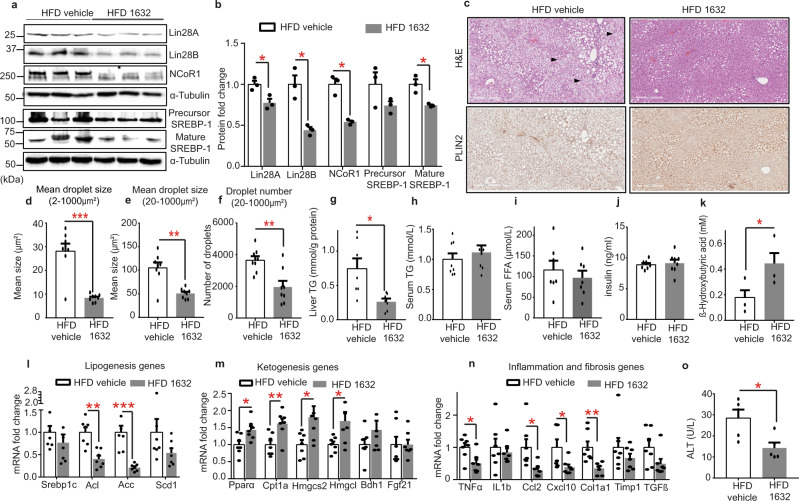
C1632 protects against high-fat diet (HFD)-induced NAFLD in mice. **a**, **b** Representative immunoblot and quantitative densitometric analyses for Lin28A, Lin28B, NCoR1 and SREBP-1 (precursor and mature form) in livers from 17-week-old male wild-type mice fed with HFD treated with vehicle or C1632 (50 mg/kg) after 4 weeks of treatment (5 IP injections per week). α-Tubulin was used as a loading control (*n* = 3 mice/group, Lin28A, *P = 0.0272; Lin28B, *P = 0.0258; NCoR1, *P = 0.0167; M-SREBP1, *P = 0.0467). **c** Representative Hematoxylin and eosin (H&E) staining and Perilipin (PLIN2) staining of liver sections from vehicle and C1632 (50 mg/kg)-treated mice fed with a HFD. The arrowheads indicate ballooned hepatocytes. Scale bars correspond to 500 μm. **d–f** Lipid droplet size and number were quantified by Image J (*n* = 8 histological images/group, **d**, ***P = 0.0004; **e**, **P = 0.0018; **f**, **P = 0.0034). **g** Hepatic triglyceride (TG) content in mice fed a HFD and treated with vehicle or C1632, as described in (**a**) (*n* = 7 mice/group; *P = 0.0175). **h–k** Serum TG *(n* = 8 mice/group), FFA (n = 7 mice/group), insulin (*n* = 7 mice/group), and β-OHB levels (*n* = 4 mice/group, *P = 0.0407) in mice fed with a HFD and treated with vehicle or C1632, as described in (**a**)**. l–n** Relative mRNA levels of lipogenesis-, ketogenesis-, inflammation- and fibrosis-related genes in livers from mice fed with a HFD and treated with vehicle or C1632 as described in (**a**) (*n* = 7 mice/group, Acl, **P = 0.0011; Acc, **P = 0.0015; Pparα, *P = 0.0184; Cpt1a, **P = 0.0087; Hmgcs2, *P = 0.0489; Hmgcl, *P = 0.0410; Tnfα, *P = 0.0160; Ccl2, *P = 0.0308; Cxcl10, *P = 0.0489; Col1a, **P = 0.0097). β-Actin was used as a housekeeping gene. **o** Serum ALT levels in mice fed with a HFD and treated with vehicle or C1632 as described in (**a**) (*n* = 5 mice/group, *P = 0.0227). Numbers to the left of the blots indicate molecular weight markers. Values are mean ± SEM. *P < 0.05; **P < 0.01; ***P  <  0.001. Groups were compared using two-tailed unpaired Student’s t-test. Source data are provided as a Source Data file.

Next, we investigated the impact of C1632 treatment on lipid metabolism in a dietary mouse model of NAFLD. NAFLD was induced by feeding 6-week-old, male wild-type mice a 40% fat, 20% fructose, and 2% cholesterol diet for 11 weeks^87,88^. During the last four weeks, mice were treated with vehicle or 50 mg/kg of C1632 5 days/week. The mice were sacrificed and their livers were isolated. Immunoblot analyses revealed reduced levels of Lin28A, Lin28B, NCoR1, and mature SREBP-1 proteins (Fig. 6a, b). Consistent with our previous data in the Pten-deficient mice, C1632 treatment reversed hepatic steatosis, as determined by reduced formation of lipid droplets and lower hepatic TG levels (Fig. 6c–g). Notably, C1632 also reduced hepatocyte ballooning, which signed progression to an early stage of steatohepatitis in this dietary model (Fig. 6c). Serum triglycerides, non-esterified fatty acids and insulin were unaffected (Fig. 6h–j) by the treatments, while ketone body production increased (Fig. 6k). We found no significant differences between the two groups of mice in total bodyweights nor liver or adipose tissue weights (Fig. S20a–c). In contrast, C1632-treated mice showed decreased hepatic expression of cholesterol and fatty acid synthesis genes, while ketogenesis and lipid oxidation genes were raised (Fig. 6l, m), in line with the suppressed lipid accumulation. The decrease in inflammation and fibrosis gene marker expression (Fig. 6n) and serum ALT levels (Fig. 6o) suggested that HFD-induced liver injury was alleviated in C1632-treated mice. Together, these results provided strong evidence that pharmacological inhibition of Lin28 protects against NAFLD and the associated pathogenic mechanisms in mice challenged with a HFD.

## Discussion

Lin28A and Lin28B are RNA-binding proteins that elicit multiple functions ranging from embryo development to tissue regeneration and tumorigenesis^1,89^. Lin28 proteins exert their activities by binding to miRNA precursors to control the biogenesis of let-7 miRNAs, or by binding the 3’UTRs of mRNAs to enhance translation^7,9,11,90,91^. A determining aspect of Lin28 biology is the Yin-Yang relationship with the let-7 miRNA family which establishes a powerful double-negative feedback loop that can switch cell states^9,14,92,93^. Pharmacological inhibition of such states can lead to amplified effects. Insights into the diverse processes regulated by Lin28 came from recognition of their impact on cell signalling, in particular the PI3K-AKT-mTOR pathway, controlling both growth and metabolism^3,15^. Therefore, Lin28 inhibition offers a means to address various disorders on the one hand, but, on the other hand, exaggerated Lin28 inhibition risks to cause mechanism-dependent toxicity. In this context, one approach to identify how such factors might be harnessed as new drug targets, is to use drug-like ligands to probe the effects of target inhibition in in vivo models. This produces a direct read-out on how a whole organism might respond to a drug action, providing information that is difficult to glean from book knowledge of affected pathways, or by using genetic methods. For example, testing analogues of rapamycin in mice and in patients has revealed that mTOR has limited value as a target for solid tumours-possibly due to feedback loops in mTOR pathways^94,95^, but may be of value for neurological disorders, thanks to its role in autophagy^96^.

Recently, we identified compound C1632 as a selective micromolar inhibitor of the Lin28/let-7 interaction^37^. This compound is being used by several groups to explore Lin28 biology^31,37,46,47,97^. Here, we showed that mice treated with C1632 exhibited moderate but transient increased blood glucose levels, concomitant with reduced levels of Lin28 and increased expression of let-7 miRNAs. We do not see prominent glucose intolerance with C1632, unlike the genetic knockout studies in mice^15,48^. Furthermore and consistent with previous reports^15,17,21,48^, Ser473 phosphorylation of AKT and INSR protein levels were reduced in skeletal muscle and liver tissue, showing that pharmacological inhibition of Lin28 suppresses signalling through the PI3K-AKT-mTOR pathway. Lin28 proteins promote insulin-mTOR signalling and lipogenesis, which are antithetical pathways to fatty acid oxidation and ketogenesis^15,32,52,58–60^. The former are activated in nutrient-affluent conditions, while ketogenesis is dominant in fasted state. In this way, the insulin-PI3K-mTOR pathway supports metabolic plasticity in differential nutritional states^52,98–100^.

We discovered that treatment of mice with C1632, triggered ketogenesis in normal-fed mice as indicated by elevated serum β-OHB and increased hepatic expression of ketogenic genes. We validated these findings in cells and confirmed that they derived from Lin28 inhibition by reproducing the effects with alternative Lin28 inhibitors. Using C1632, we sought to identify the molecular mediators linking the Lin28/let-7 axis with ketogenesis. Ketogenesis is regulated via mTORC1 signalling in liver by NCoR1^52,59^, an energy sensor that represses the major activator of ketogenesis PPARα. The activity and selectivity of NCoR1 protein is regulated during ketogenesis by differential phosphorylation status^58^ and by autophagy^69^. We identified a conserved binding site for let-7 in the 3′UTR of NCoR1 mRNA and demonstrated that increased let-7 activity induced by C1632 treatment also suppresses NCoR1 protein. This, in turn, contributes to the release of a ketogenesis gene expression program driven by PPARα and leads to increased expression of ketogenic genes.

Nonalcoholic fatty liver disease (NAFLD) is characterized by excessive hepatic lipid accumulation (steatosis) and impaired ketogenesis^73,101^. Hepatic lipid content results from the integration of counteracting processes, including lipogenesis and fatty acid oxidation/ketogenesis. Enhancing the latter, reduces lipid load in the liver and is a promising therapeutic approach to manage NAFLD^56,66,102–104^ for example, via PPARα-agonists^105,106^. Lin28 proteins promote lipogenesis by controlling translation of the master regulator of lipid synthesis SREBP-1^32^, whereas genetic ablation of Lin28B reduces liver steatosis and fibrosis in mice with alcoholic liver injury^107^. Modulation of insulin-AKT signalling is likely to be one of the mechanisms by which Lin28 regulates hepatic lipid metabolism. The insulin-AKT-mTOR signalling network consists of many molecular components that add complexity to the system, which at least partly may explain conflicting data regarding the regulation of lipid metabolism by mTORC^53,108–110^. However, several studies show that the insulin-AKT-mTOR pathway promotes the function of SREBP1 and aggravates liver steatosis^80,100,109,111–115^. Interestingly, a recent study by Gosis et al.^115^ has now demonstrated that selective inhibition of mTORC1 downstream effectors can protect mice from NAFLD^115^.

We found that C1632 treatment of AML12 and HepG2 cells decreased SREBP1, mRNA levels of fatty acid synthetic enzymes, and lipid accumulation. Hence, we propose that the effect of C1632 on lipid deposition results from repression of lipogenesis in concert with increased lipid breakdown through activation of fatty acid oxidation and ketogenesis, with the latter dependent on direct intervention of let-7 miRNAs on the NCoR1/PPARα co-repressor complex. NCoR1 is a corepressor of many nuclear receptors, not only PPARα and lipogenic pathways are up-regulated in NCoR1 KO mice^58^. Based on this mechanism, the antisteatotic effect of pharmacological Lin28 inhibition seems paradoxical. However, it is plausible that the effect of C1632 on the lipogenic transcription factor SREBP1 might simply be the principal driver of the C1632-mediated lipogenesis inhibition.

Our observations in hepatocyte cell lines motivated us to investigate the effect of C1632 in two distinct preclinical mouse models of NAFLD, including liver-specific Pten-deficient mice^78,79^ and mice fed with a NAFLD-diet. Importantly, C1632 treatment protected against NAFLD in both mouse models, as evidenced by reduced hepatic steatosis, improved lipid homeostasis, attenuated inflammation and liver damage. We carried out the study in male mice, because of the more severe steatosis that they develop. Further studies can be performed to evaluate if the metabolic effects of 1632 are gender-independent.

The inhibition of Lin28 activity has gained attention as a therapeutic approach to reprogram cellular metabolism in different disease mechanisms^30,31^. Research so far has mostly focused on intervention in glucose metabolism to target cancer or diabetes^15,17,31,47^. Interestingly, ketone bodies have been shown to decrease cancer cell growth and promote patient survival in several types of cancer^50,116^. Our work with C1632 suggests that pharmacological inhibition of Lin28 is a possible alternative means to dietary modification and may provide a controlled intervention for the treatment of NAFLD/NASH through increased ketogenesis. The next logical step would be medicinal chemistry programs aimed at advancing more potent and more selective Lin28 inhibitors.

## Methods

### Animals and treatments

Study protocols were approved by the Swiss Veterinary Authority (license number:31966). Mice were obtained by commercial sources (C57BL/6J; Crl:NU(NCr)-Foxn1nu, Charles River Laboratories) or generated in house (Alb-Cre;Ptenflox/flox). Animals were housed in pathogen-free conditions on a 12 h light/dark cycle at 20-24 °C and 50-65% relative humidity. During the experiments, animal health state was monitored three times a week for signs of discomfort. The humane endpoints were based on a scoring system described in the mouse experiment guidelines approved by the Swiss Veterinary Authority (license number: 31966). Signs of dehydration, body weight and body conditions (including posture and vertebral appearance) were scored and recorded. Additionally, behaviour and physical conditions, including hair coat, eyes, nose, mouth, genitals and skin were scored and recorded. Euthanasia was performed by CO2 inhalation. All experiments were performed with age-matched male mice (male Alb-Cre;Ptenflox/flox mice develop more severe steatosis compared with females). Compound C1632 was stored at 4 °C and dissolved in 12.5% DMSO immediately before administration. Mice were randomly assigned to receive vehicle (12.5% DMSO) or C1632 (50 mg/kg) via intraperitoneal (IP) injections. Treatment schedules were the following: a) daily for 5 days (7-week-old, male C57BL/6J and 7-week-old male Crl:NU(NCr)-Foxn1nu) and b) 3 times/week for 3 weeks (7-week-old male Crl:NU(NCr)-Foxn1nu) or 4 and 6 weeks (6- and 22-week-old male Alb-Cre;Ptenflox/flox). For the C1632 treatment in the dietary mouse model, 6-week-old male wild-type mice were fed a Rodent Diet with 40 kcal% fat (mostly palm oil), 20 kcal% fructose and 2% cholesterol supplied from ResearchDiet (#D09100310) or chow diet (3432 Maintenance, KLIBA NAFAG cod. 3432. PX.V20) for 11 weeks. During the last 4 weeks of the NAFLD diet mice were randomly assigned to receive vehicle (12.5% DMSO) or C1632 (50 mg/kg) via intraperitoneal (IP) injections (5 days/week). Blood glucose measurements were taken from tail vein blood using glucometer from Axapharm–Healthpro and test strips. Ketonaemia was measured using an Abbot–Freestyle device. For Glucose Tolerance Test (GTT) and Insulin Tolerance Test (ITT), mice (4-5/group, C57BL/6J and Crl:NU(NCr)-Foxn1nu) were randomly assigned to overnight fast or normal diet and to receive vehicle or C1632 injections. At 30 min post-treatment, fasted mice were injected with glucose (2 mg/g body weight, IP) or Insulin (0.75 U /kg body weight, IP). Blood samples were taken before and at 30, 60, and 120 min after last injection. At the end of each experiment, mice were euthanized after 3 h from the last treatment. Blood was collected by cardiac puncture into capillary blood collection tubes with coagulation inducer and separation gel (GK 150 SE 200 µL Gel, KABE Labortechnik). Serum was separated by centrifugation (5 min at 5,600 rpm). Organs, such as liver and muscle were collected and snap-frozen or formalin-fixed for molecular, immune-histochemical and histopathological analyses.

### Compounds

N-methyl-N-[3-(3-methyl[1,2,4]triazolo[4,3-b]pyridazin-6-yl)phenyl]acetamide (C1632) was purchased from Maybridge (SB02011EA). N-(4-(N-(3-(4-benzylpiperazin-1-yl)propyl)sulfamoyl)−2-chlorophenyl)acetamide (C4019) was synthesized as previously described^37^ and analysed by NMR spectroscopy. 4-(7-(4-((benzyloxy)carbonyl)piperazin-1-yl)−4,4-dimethyl-8-nitrochromeno[4,3-c]pyrazol-1(4H)-yl)benzoic acid (C1243) was generously provided by Storm Therapeutics. Rapamycin was purchased from LC Laboratories (R-5000).

### Immunohistochemistry

Tissue samples were fixed in 10% formalin (Thermo Scientific, 5701) overnight and consequently embedded in paraffin. Sections were cut (4 μm) for immunohistochemistry and for Hematoxylin (Diapath, C0303) and Eosin (Diapath, C0363) staining. Once dried, sections were treated with OTTIX plus solution (Diapath, X0076) and OTTIX shaper solution (Diapath, X0096) for dewaxination and rehydration. Antigen retrieval was performed using pH 6 sodium citrate at 98 °C for 20 min. Successively, endogenous peroxidases were blocked using 3% H2O2 (VWR chemicals, 23615.248), and non-specific binding sites were blocked with Protein-Block solution (DAKO Agilent technologies, X0909) for 10 min. After antigen retrieval, sections were incubated with anti-Perilipin 2 (Progen GP46, 1:800) antibody at room temperature for 1 h and followed by secondary antibody anti-Guinea Pig (Adipogen AG, 1:400) incubation for 30 min. Perilipin 2 staining was used to quantify number and size of lipid droplets using ImageJ.

### Cell culture, plasmid transfection and compound treatment

Human HepG2 (HB-8065), AML12 (CRL-2254), HEK293T (CRL-3216) and mouse C2C12 (ECACC 91031101) cell lines were obtained from American Type Culture Collection (ATCC). HepG2 cells were maintained in DMEM/F-12 GlutaMAXTM (Gibco) supplemented with 10% of FBS and 1% antibiotics (Penicillin/Streptomycin). AML12 cells were grown in DMEM/F12 Medium, supplemented with 10% FBS, 10 µg/ml insulin, 5.5 µg/ml transferrin, 5 ng/ml selenium, 40 ng/ml dexamethasone, at 37 °C and 5% CO2. HEK293T cells were grown in DMEM (Gibco) supplemented with 10% foetal bovine serum (FBS). Mouse C2C12 myoblasts were grown as undifferentiated myoblasts in DMEM (Gibco) supplemented with 10% foetal bovine serum (FBS), 1.5% GlutaMax and 1% antibiotics (Penicillin/Streptomycin), at 37 °C and 5% CO_2_. To induce differentiation of C2C12 myoblasts to myotubes, cells were allowed to reach 80-90% confluence and the media was switched to differentiation media, DMEM supplemented with 2% horse serum, 1.5% GlutaMax and 1% antibiotics (Penicillin/Streptomycin). The transfections were performed according to the manufacturer’s protocol with Lipofectamine 2000 (Invitrogen). Plasmid transfections in HepG2 cells were performed with Lipofectamine 3000 (Invitrogen). Cells were transfected with the following plasmids: pCMV6-XL5 and pCMV-cMYC-LIN28 (Origene), pBABE-zeo (pBABE-bleo), pBABE-LIN28B (Addgene), mouse NCoR1 ORF (Sino Biological). C2C12 cells were transfected on the 6^th^ day of differentiation. For plasmid transfection in HepG2 cells, 8 × 10^5^ cells were seeded in each well of a 6-well plate and the cells were transfected 1 day following cell seeding. For cellular treatment, N-methyl-N-[3-(3-methyl[1,2,4]triazolo[4,3-b]pyridazin-6-yl)phenyl]acetamide (C1632) was dissolved in 10% DMSO, resulting in a concentration of 0.15% DMSO content in the cell growth media. N-(4-(N-(3-(4-benzylpiperazin-1-yl)propyl)sulfamoyl)−2-chlorophenyl)acetamide (C4019), and 4-(7-(4-((benzyloxy)carbonyl)piperazin-1-yl)−4,4-dimethyl-8-nitrochromeno[4,3-c]pyrazol-1(4H)-yl)benzoic acid (C1243) were dissolved in DMSO, resulting in a final DMSO concentration of 1.5% for C4019 and 0.4% for C1243. Rapamycin was diluted in DMSO, resulting in a concentration of 0.0003% DMSO content in the cell growth media. Cells treated with indicated concentrations of compounds were treated with equal volumes of DMSO. C2C12 cells were seeded in a 6-well plate (10^6^ cells/well) and they were treated with compounds, while being in differentiation medium. HepG2 cells were seeded in a 6-well plate (8 × 10^5^ cells/well) and they were treated with C1632 or C4019 one day after cell plating. For the β-OHB measurement, cells were subjected to a long-chain Free Fatty Acid mixture (oleic acid (ABCR GMBH): palmitic acid (Sigma-Aldrich), 2:1) at a final concentration of 0.9 mM to increase the ketogenic substrates.

### siRNA and pre-let-7 synthesis and transfection

SiRNA targeting Lin28b, scramble sequence, pre-miR-16-2, pre-let7a1, pre-let7c, pre-let7d, pre-let7e and pre-let7g, were synthesized with a MM12 synthesizer from Bio Automation Inc. (Plano, TX) on 500 Å or 2000 Å Unylinker CPG from ChemGenes (Wilmington, MA). Chemicals for oligonucleotide synthesis were purchased from Aldrich and TCI (Sigma-Aldrich Chemie GmbH, D-89555 Steinheim) and phosphoramidites were obtained from Thermo Fisher Scientific (Waltham, MA), Glen Research (SterLing, VA). The coupling time for phosphoramidites was 2x90s. The RNA phosphoramidites were prepared as 0.08 M solutions in dry acetonitrile (ACN), the activator BTT (Biosolve BV, 5555 CE Valkenswaard, the Netherlands) was prepared as a 0.24 M solution dry ACN, 5-(Benzylthio)−1H-tetrazole (Carbosynth) was used at 0.24 M concentration in dry acetonitrile to activate the phosphoramidites for coupling. Oxidizer was prepared as a 0.02 M I2 solution in THF/Pyridine/H2O (70:20:10, w/v/v/v); the sulfurizing reagent was prepared as a 0.05 M solution of 3-((N,N-dimethylaminomethylidene)amino)−3H-1,2,4-dithiazole-5-thione (DDTT; Sulfurizing Reagent II; Glen Research, Virginia) in dry pyridine/ACN (60:40). Capping reagent A was: THF/lutidine/acetic anhydride (8:1:1) and capping reagent B was: 16% N-methylimidazole/THF. Deblock solution was prepared as a 3% dichloro acetic acid in dichloromethane. The cleavage, the bases and phosphodiester backbone deprotection was done by incubation of the CPG support for 45 min, at 65 °C in AMA solution (conc. ammonia:methylamine; 1:1). Deprotection of 2’-O-TBDMS (tertbutyldimethylsilyl-) was performed by incubation of the oligonucleotides for 1.5 h, at 70 °C in a mixture of dry 1-N-methyl-2- pyrrolidone/triethylamine/triethylamine trihydrofluoride (6/3/4). The oligonucleotides were purified DMT-on and DMT-off by RPHPLC on an Agilent 1200 series preparative HPLC fitted with a Waters XBridge OST C-18 column, 10 × 50 mm, 2.5 μm at 60 °C. Running buffer for HPLC purification of single-stranded ORNs (up to 23 nt): buffer A (0.1 M triethylammonium acetate), buffer B (acetonitrile); gradient for the DMT-on purification: 20–50% buffer B over 5 min; gradient for the DMT-off purification: 5 –35% buffer B over 5 min. Fractions containing the product were collected and dried in a miVac duo SpeedVac from Genevac, diluted with water to 20 µM concentration. Mass and purity (>95%) was confirmed by LCMS (Agilent 1200/6130 system) on a Waters Acquity OST C-18 column, 2.1 × 50 mm, 1.7 mM, 65 °C. Buffer A: 0.4 M HFIP, 15 mM triethylamine; buffer B: MeOH. Gradient: 7–35% B in 12 min; flow rate: 0.3 ml/min. The oligonucleotide sequences are as follows:

Guide and passenger sequences of phosphodiester oligoribonucleotides (ORN) for siLin28B were 5’-AAAUCCUUCCAUGAAUAGUTT-3’ and 5’-ACUAUUCAUGGAAGGAUUUTT-3’, respectively.

SiRNA negative control: 5′-GUGXXUAAXXAACXCAXAC-3 and 5-GUGUGUGUUGUUUAUGCAC-3

Pre-miR16-2: 5′UAGCAGCACGUAAAUAUUGGCGUAGUGAAAUAUAUAUUAAACACCAAUAUUACUGUGCUGCUUUA-3′

Pre-let7a1: 5′-UGAGGUAGUAGGUUGUAUAGUUUUAGGGUCACACCCACCACUGGGAGAUAACUAUACAAUCUACUGUCUUUC-3′

Pre-let7c: 5′-UGAGGUAGUAGGUUGUAUGGUUUAGAGUUACACCCUGGGAGUUAACUGUACAACCUUCUAGCUUUCC-3′

Pre-let7d: 5′-AGAGGUAGUAGGUUGCAUAGUUUUAGGGCAGGGAUUUUGCCCACAAGGAGGUAACUAUACGACCUGCUGCCUUUCU-3′

Pre-let7e: 5′-UGAGGUAGGAGGUUGUAUAGUUGAGGAGGACACCCAAGGAGAUCACUAUACGGCCUCCUAGCUUUCC-3′

Pre-let7g: 5′-UGAGGUAGUAGUUUGUACAGUUUGAGGGUCUAUGAUACCACCCGGUACAGGAGAUAACUGUACAGGCCACUGCCUUGC-3′

SiRNAs and pre-let-7 oligonucleotides were transfected into HepG2 and C2C12 cells by using Lipofectamine 2000 (Invitrogen) according to the manufacturer’s instructions. SiRNA was transfected at a final concentration of 20 nM while 2.5 µg of pre-microRNAs were used for let-7 overexpression in 6-well plates. HepG2 cells were seeded 24 h before transfection at a density of 4 × 10^5^ and 8 × 10^5^ for siRNA and let-7 precursors transfections respectively.

### Protein extraction and immunoblot analysis

Mouse tissues were excised rapidly and snap-frozen in liquid N_2_. Liquid nitrogen and a mortar and pestle were used to grind the tissue samples while they were still frozen. Tissues were homogenized in RIPA buffer (Sigma), supplemented with a mixture of protease inhibitors (Roche) and PhosSTOP (Roche) using a TissueLyser (Qiagen). Cells were lysed in ice-cold RIPA lysis buffer, (150 mM NaCl, 1.0% IGEPAL CA-630, 0.5% sodium deoxycholate, 0.1% SDS, 50 mM Tris, pH 8.0, proteinase inhibitor cocktail (Roche) and PhosSTOP (Roche)), incubated for 20 min on ice and centrifuged at 20,000 × *g* for 20 min at 4 °C. Protein concentration was determined by Pierce protein assay BCA (Thermo Scientific). Equal amounts of protein were heat-denaturated in Laemmli buffer for 5 min at 95 °C. For western blot analysis, extracted proteins were resolved by 10% polyacrylamide gel and transferred to a nitrocellulose membrane (Amersham Protran). Membranes were blocked in TBST buffer (Tris-buffered saline, 0.1% Tween 20) supplemented with 5% nonfat dry milk for 1 h at room temperature and then incubated with primary antibodies overnight at 4 °C. Membranes were washed three times for 5 min and incubated with KPL Peroxidase-Labelled anti-mouse or anti-rabbit secondary antibodies (SeraCare) for 1 h at room temperature. Blots were washed three times and developed with ECL Prime Western Blotting Detection Reagent (GE Healthcare- RPN 2232). Primary antibodies used were: anti-Lin28 (#8706, Cell Signaling; 1:1000), anti-Lin28b (#4196, Cell Signaling; 1:1000), anti-Lin28b (mouse preferred) (#5422, Cell Signaling; 1:1000), anti-insulin receptor beta (sc-57342, Santa Cruz; 1:1000), anti-total-Akt (#4691, Cell Signaling; 1:1000), anti-phospho-Akt Ser473 (#4060, Cell Signaling; 1:1000), anti-α-Tubulin (sc-5286, Santa Cruz; 1:1000), anti-NCoR1 (20018-1AP, Proteintech; 1:1000), anti-NCoR1 (#5948, Cell Signalling; 1:1000), anti-β-Actin (sc-47778, Santa Cruz; 1:1000), anti-Pten (sc-7974, Santa-Cruz; 1:1000), anti-PPARα (66826-1-Ig, Proteintech; 1:2000), anti-GAPDH (60004-1-Ig, Proteintech; 1:50000), anti-SREBP1 (66875-1-Ig,Proteintech, 1.2000),anti-Phospho-p44/42 MAPK (Erk1/2) (Thr202/Tyr204) (#4377, Cell Signaling; 1:1000), anti-p44/42 MAPK (Erk1/2) (#9102, Cell signalling; 1:1000), anti-PS6Ser235 (67898-1-Ig,Proteintech; 1:5000), anti-S6 (14823-1-AP, Proteintech; 1:1000), anti-Phospho-Insulin Receptor β (Tyr1361) (#3023,Cell Signalling; 1:1000). Densitometry analysis of the gels was carried out using ImageJ software. Uncropped and unprocessed scans of the blots are supplied in the Source Data file or as supplementary figures in the Supplementary Information.

### RNA isolation, real-time quantitative PCR and Taqman PCR

Total RNA was extracted with Trizol reagent (Invitrogen) and treated with DNase (Qiagen) according to the manufacturer’s instructions. 1 μg of total RNA was reverse transcribed in 20-μl reaction mixtures with M-MLV reverse transcriptase, random hexamer primers and oligo(dT) (Thermo Fisher). The SYBR Green PCR was performed in a LightCycler 480 Real-Time PCR system (Roche) with KAPA SYBR® FAST Master Mix (KAPA BIOSYSTEMS). Each reaction was carried out in three technical replicates and β-Actin was used as a reference to normalize mRNA levels. The sequences of the primers used for the analysis of mouse livers, AML12 and HepG2 cells are provided in Supplementary Table 1. Taqman PCR was performed with standard reagents from Life Technologies (Taqman micro-RNA assays: hsa-let-7a: 000377, hsa-let-7b: 002619, hsa-let-7e: 002406, hsa-miR-16: 000391, hsa-let-7c: 000379, hsa-let-7g:002282 and U6snRNA: 001973). The Reverse Transcription was performed using the TaqMan primers from MicroRNA Assays and the TaqMan MicroRNA Reverse Transcription Kit (Thermo Fisher) with 25 ng total RNA. U6snRNA was used as an internal control. The Taqman PCR was performed in a LightCycler 480 instrument (Roche) with GoTaq Probe qPCR Master mix (Promega) according to the manufacturer’s protocol. Each reaction was carried out in three technical replicates.

### Plasmid cloning

NCoR1 reporter plasmids were generated by cloning target site inserts, synthesized by Microsynth (Balgach, Switzerland), into psiCHECK-2 vector (no. C8021, Promega, Dübendorf). Sequences of inserted target sites were as follows:

NCoR1 WT 5’−3’:

CTC GAG AGG CTT ATT TGA GGC ATA CCT CAC TGT TAC GCA CAC TGG GC

NCoR1 MUT 5’−3’:

CTC GAG AGG CTT ATT TGA GGC AAA GCA GAC TGT TAC GCA CAC TGG GC

### Luciferase assay

The putative *let-7* target site in the 3'UTR of NCoR1 or its mutated form were inserted into psiCHECK-2 dual-luciferase reporter vectors. HEK293T cells were seeded in opaque white 96-well-plates (Roskilde) in 80 μl medium*/*15.000 cells per well. After 8 hours, cells were transfected in technical replicates with pre-let-7a, pre-let-7c or negative control RNA at 40 nM, using Lipofectamine 2000 (Invitrogen). After 24 h from RNA transfection, cells were transfected with psiCHECK-2 dual-luciferase reporter vectors, using JetPEI (Polyplus) at 20 ng/well. After 48 hours from plasmid transfection, luciferase activity was measured according to the manufacturer’s protocol (Dual-Glo Luciferase Assay System, Promega) with 30 μl/well Dual-Glo® Luciferase Reagent diluted in 1:1 ratio with H2O, and 15 μl/well of Dual-Glo® Stop & Glo® Reagent. Readout was performed on a microtiter plate reader (Mithras LB940, Berthold Technologies). Values were normalized to the firefly luciferase counts and additionally to the corresponding values obtained from negative control treatment. To examine let-7 activity after C1632 treatment, we treated HEK293-T cells with 0, 25, 50, 100 μΜ C1632 and 24 h later, cells were transfected with reporter plasmid. Luciferase activity was measured as above described.

### 2-NBDG glucose uptake assay

HepG2 cells were seeded in six-well plates (8 × 10^5^ cells/well) in DMEM/F-12 GlutaMAXTM (Gibco) supplemented with 10% of FBS and 1% antibiotics (Penicillin/Streptomycin). Cells were treated with 100 μΜ of C1632 for 4 days. On the day of the flow cytometry assay cells were incubated in glucose-free DMEM medium (Gibco) at 37 °C for 2 h. Cells were then incubated with 200μΜ of 2-[N-(7-nitrobenz-2-oxa-1,3-diazol-4-yl)amino]−2-deoxy-D-glucose (2-NBDG) (Biovision) at 37 °C for 45 min. Cells were washed with phosphate-buffered saline (PBS) and subsequently detached using trypsin for flow cytometry analysis. Cells were washed with 1 ml PBS prior to resuspension in 200 µl FACS buffer (2% FBS in PBS) and subsequently strained through CellTrics 50μm filters. For each measurement, data from 20 × 10^3^ single viable cell events were acquired using a Flow cytometer (BD LSR Fortessa) using the blue laser (excitation: 488 nm) with emission at 530/30 nm (FL-2 photomultiplier). The gating strategy was designed to exclude dead cells and cell doublets. Data were analysed with FlowJo software (BD Biosciences).

### Oil Red O staining

HepG2 cells were plated on glass coverslips in 6 wells-plates (8 × 10^5^ cells/well). After 24 h, a group of cells was treated with C1632 (0, 50, 100 and 150 µM). The second group of cells was treated with a single dose of Oleic acid (0.1 mM) and C1632 (0, 50, 100 and 150 µM). The third group of cells was treated with 5 daily doses of Oleic acid (0.1 mM) and C1632 (0, 50, 100 and 150 µM). All groups were fixed after a week and processed for staining using an Oil Red O Stain Kit (abcam, ab150678) according to the manufacturer’s instructions. AML12 cells were plated in 6-well plates (4 × 10^5^ cells/well). A group of cells was treated with vehicle, the second group of cells was treated with vehicle and 3 daily doses of Oleic Acid (0.1 mM), and the third group of cells was treated with 3 daily doses of Oleic Acid (0.1 mM) and C1632 (100 µM). All groups were fixed with 4% formalin, followed by two washes with water and incubation with 60% isopropanol. 60% isopropanol was discarded and cells were covered with Oil Red O solution. Cells were washed with water and viewed under microscope. Lipid content was quantified based on the intensity of Oil Red O staining in cells using ImageJ.

### β-Hydroxybutyric (β-OHB) acid measurement

β-OHB levels in mouse serum, HepG2 or AML12 growth medium were determined using a spectrophotometric enzymatic assay (MAK134, Sigma) according to the manufacturer’s protocol. Total protein was also isolated from each well and used to normalize β-OHB levels in the growth medium of HepG2 cells. To remove interfering substances, growth medium samples were filtered with Amicon Ultra-0.5 Centrifugal Filter Units (UFC501096) prior to measurement. The optical density was assessed on a SpectraMax Paradigm plate reader (Molecular Devices) at 340 nm.

### Measurement of lactate production

The extracellular lactate was measured using a colorimetric assay (MAK064, Sigma) following the manufacturer’s protocol. The values were normalized to the total protein concentration of the samples. The optical density was assessed on a SpectraMax Paradigm plate reader (Molecular Devices) at 570 nm.

### Alanine aminotransferase activity assay

The levels of ALT in serum were determined using a commercial colorimetric assay kit from Elabscience (E-BC-K235-M). ALT activity was calculated by measuring the OD values at 510 nm on a SpectraMax Paradigm plate reader (Molecular Devices).

### Triglyceride (TG) colorimetric assay

The preparation of liver tissue samples and the measurement of hepatic triglyceride were done according to the manufacturer instructions in a colorimetric triglyceride assay kit from Elabscience (E-BC-K238). The absorbance of the samples was measured at 510 nm on a SpectraMax Paradigm plate reader (Molecular Devices). The protein concentration of liver homogeneates was determined to calculate the TG content in liver tissues.

### Analysis of serum insulin levels

Serum insulin levels from mice were measured by Enzyme Linked Immunosorbent Assay (ELISA). The analysis was conducted using Mouse Insulin Elisa Kit (Elabscience, China). The procedure was performed according to the manufacturer’s instructions.

### Free fatty acids (FFA) fluorometric assay

The non-esterified free fatty acids (FFA) content in serum samples was measured by the FFA assay kit (Elabscience Biotechnology) according to the manufacturer’s instructions. Fluorescence emission at 590 nm was collected with excitation at 535 nm in a microplate reader (Tecan Spark 20 M).

### Statistical analysis

Data are shown as means ± standard error of the mean (SEM). Statistical analysis was performed using the unpaired two-tailed Student’s t-test and two-way ANOVA with fisher’s least significant difference post hoc analysis or one-way ANOVA with Bonferroni’s post hoc test as indicated. Results were considered significant at *P*  <  0.05. GraphPad Prism Software 7 (GraphPad) was used to produce the graphs and perform the statistical analysis.

### Reporting summary

Further information on research design is available in the Nature Portfolio Reporting Summary linked to this article.

## Supplementary information

Supplementary Information

Reporting Summary

## Data Availability

All data generated or analysed during this study are included in this published article (and its supplementary information files). Source data are provided with this paper.

## Source data

Source Data
