# Supplementary material for: Pharmacological inhibition of Lin28 promotes ketogenesis and restores lipid homeostasis in models of non-alcoholic fatty liver disease
Source: Nat Commun. 2022 Dec 26;13:7940. doi: 10.1038/s41467-022-35481-1 (PMC9792516; doi:10.1038/s41467-022-35481-1)
Supplement: Supplementary file 1 — Supplementary Information [file 41467_2022_35481_MOESM1_ESM.pdf]

Supplementary Information

Pharmacological inhibition of Lin28 promotes ketogenesis and restores lipid homeostasis in models of non-alcoholic fatty liver disease

Evangelia Lekka[1], Aleksandra Kokanovic[2], Simone Mosole[2], Gianluca Civenni[2], Sandro Schmidli[1], Artur Laski[1], Alice Ghidini[1], Pavithra Iyer[1], Christian Berk[1], Alok Behera[1], Carlo V. Catapano*[2], Jonathan Hall*[1].

[1]Department of Chemistry and Applied Biosciences, Institute of Pharmaceutical Science, ETH Zurich, Zurich, Switzerland

[2]Tumor Biology and Experimental Therapeutics, Institute of Oncology Research (IOR) Università della Svizzera Italiana (USI), Bellinzona, Switzerland

Table of Contents

Supplementary Information ***1***

Supplementary Figure 1. Densitometric analyses for immunoblots of Figure 1. .... **3**

Supplementary Figure 2. 1632 treatment does not affect serum insulin levels in mice. **5**

Supplementary Figure 3. 1632 decreases Insulin receptor tyrosine 1361 phosphorylation in cells. **6**

Supplementary Figure 4. Lin28B inhibition results in decreased lactate secretion in HepG2 cells. **7**

Supplementary Figure 5. 1632 treatment did not change body weight in mice. **8**

Supplementary Figure 6. 1632 treatment increases circulating ketone body levels in nude mice. **9**

Supplementary Figure 7. 1632 Treatment caused a moderate glucose intolerance in wild type mice but did not alter blood glucose levels in nude mice. **10**

Supplementary Figure 8. 1632 treatment increased expression of ketogenic genes in livers of nude mice. **11**

Supplementary Figure 9. Densitometric analyses for immunoblots in Fig.3 .... **12**

Supplementary Figure 10. NCoR1 overexpression abrogates the 1632-mediated ketogenesis activation. **13**

Supplementary Figure 11. Let-7 overexpression validation in let-7-transfected HepG2 cells. 14

Supplementary Figure 12. Let-7c represses levels of NCoR1 mRNA. 15

Supplementary Figure 13. 1632 treatment inhibits Lin28B in HEK293T cells. 16

Supplementary Figure 14. Transfection of HepG2 cells with let-7 induces expression of PPARα.17

Supplementary Figure 15. Densitometric analyses for immunoblots in Figure 4. 18

Supplementary Figure 16. Liver-specific Pten$^{-/-}$ male mice develop fatty liver phenotype. 19

Supplementary Figure 17. C1632 Treatment in 10-wk old liver-specific Pten$^{-/-}$ male mice. 20

Supplementary Figure 18. 1632 decreases inflammation and fibrosis markers in Pten-deficient livers. 21

Supplementary Figure 19. 1632 Treatment does not alter liver/body weight in 28-wk old Pten$^{-/-}$ mice. 22

Supplementary Figure 20. Mouse, liver, and adipose tissue weights are not significantly different between vehicle- and 1632-treated mice on HFD. 23

Supplementary Figure 21. Full blots for Supplementary Figure.3 24

Supplementary Figure 22. Full blots for Supplementary Fig. 8b,d 25

Supplementary Figure 23. Full blots for Supplementary Figure 10 25

Supplementary Figure 24. Full blots for Supplementary Figure 13. 26

Supplementary Figure 25. Full blots for Supplementary Figure 14. 27

Supplementary Figure 26. Full blots for Supplementary Figures 16e and 17e 28

Supplementary Figure 27. The gating strategy for 2-NBDG glucose uptake assay. 29

Supplementary Figure 28. LCMS chromatograms of oligonucleotides. 32

Supplementary Table 1 33

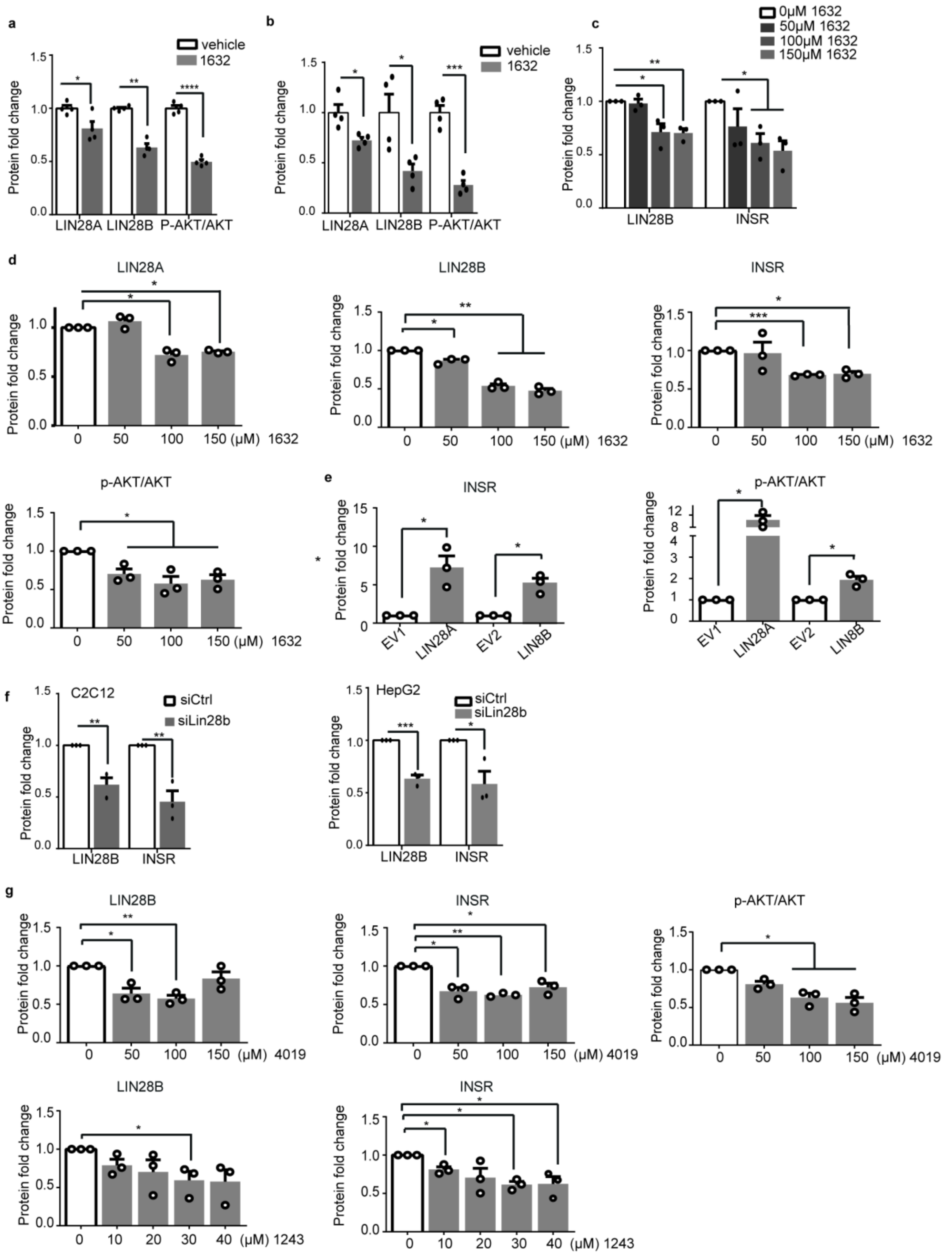

Supplementary Figure 1. Densitometric analyses for immunoblots of Figure 1.

a)-g) Protein quantification for immunoblots shown in **Figs. 1c** (n=4mice/group; Lin28A, P=0.0388; Lin28B, P=0.0012; P-AKT/AKT, P<0.0001), **1d** (n=4 mice/group; Lin28A, P=0.0338; Lin28B, P=0.0444; P-AKT/AKT, P=0.0003)**, 1f** (n=3 biologically independent experiments; Lin28B, 0 vs 100μM, P=0.0220; 0 vs 15 0μM, P=0.0019; INSR, 0 vs 100 μM, P=0.0116; 0 vs 150 μM, P=0.0390)**, 1j** (n=3 independent experiments; Lin28A; 0 vs 100 μM, P=0.0173; 0 vs 150 μM, P=0.0013; Lin28B, 0 vs 50 μM, P=0.0253; 0 vs 100 μM, P=0.0033; 0 vs 150 μM, P=0.0029; INSR, 0 vs 100 μM, P=0.0005; 0 vs 150 μM,

P=0.0103)**,** P-AKT/AKT, 0 vs 50 μM, P=0.0461; 0 vs 100 μM, P=0.0461; 0 vs 150 μM, P=0.0295) **1k** (n=3 independent experiments, INSR; EV1 vs Lin28A, P=0.0137; EV2 vs Lin28B, P=0.0281; P-AKT/AKT, EV1 vs Lin28A, P=0.0224; EV2 vs Lin28B, P=0.0311)**, 1l** (n=3 independent experiments, (C2C12) Lin28B, siCtrl vs siLin28B, P=0.0049; INSR, siCtrl vs siLin28b, P=0.0068; (HepG2), Lin28B, siCtrl vs siLin28b, P=0.0005; INSR, siCtrl vs siLin28b, P=0.0267)**, 1m** (n=3 independent experiments; (4019) Lin28B, 0 vs 50 μM, P=0.0359; 0 vs 100 μM, P=0.0099; INSR, 0 vs 50 μM, P=0.0230; 0 vs 100 μM, P=0.0013; 0 vs 150 μM, P=0.0342; P-AKT/AKT, 0 vs 50 μM, P=0.0368; 0 vs 100 μM P=0.0152; 0 vs 150 μM P=0.0248; (1243), Lin28B, 0 vs 30 μM P=0.0257; INSR, 0 vs 10 μM P=0.0330; 0 vs 30 μM P=0.0109; 0 vs 40 μM P=0.0173). Values are mean ±SEM. *P < 0.05; **P < 0.01, ***P < 0.001, ****P < 0.0001. Groups were compared using two-tailed unpaired Student's t-test.

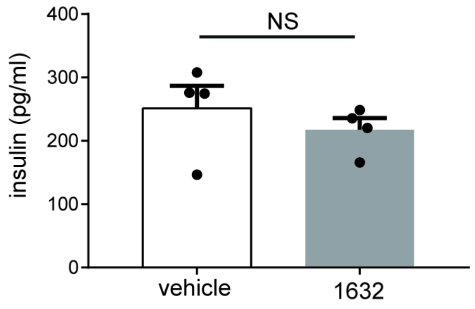

Supplementary Figure 2. 1632 treatment does not affect serum insulin levels in mice.

Serum insulin levels in 7-week-old C57BL/6J wild-type mice after intraperitoneal (IP) injections of 1632 (50mg/kg) or vehicle for 5 consecutive days, while mice were fed *ad libitum* (n=3 mice/group). Values are mean ±SEM. Groups were compared using two-tailed unpaired Student's t-test.

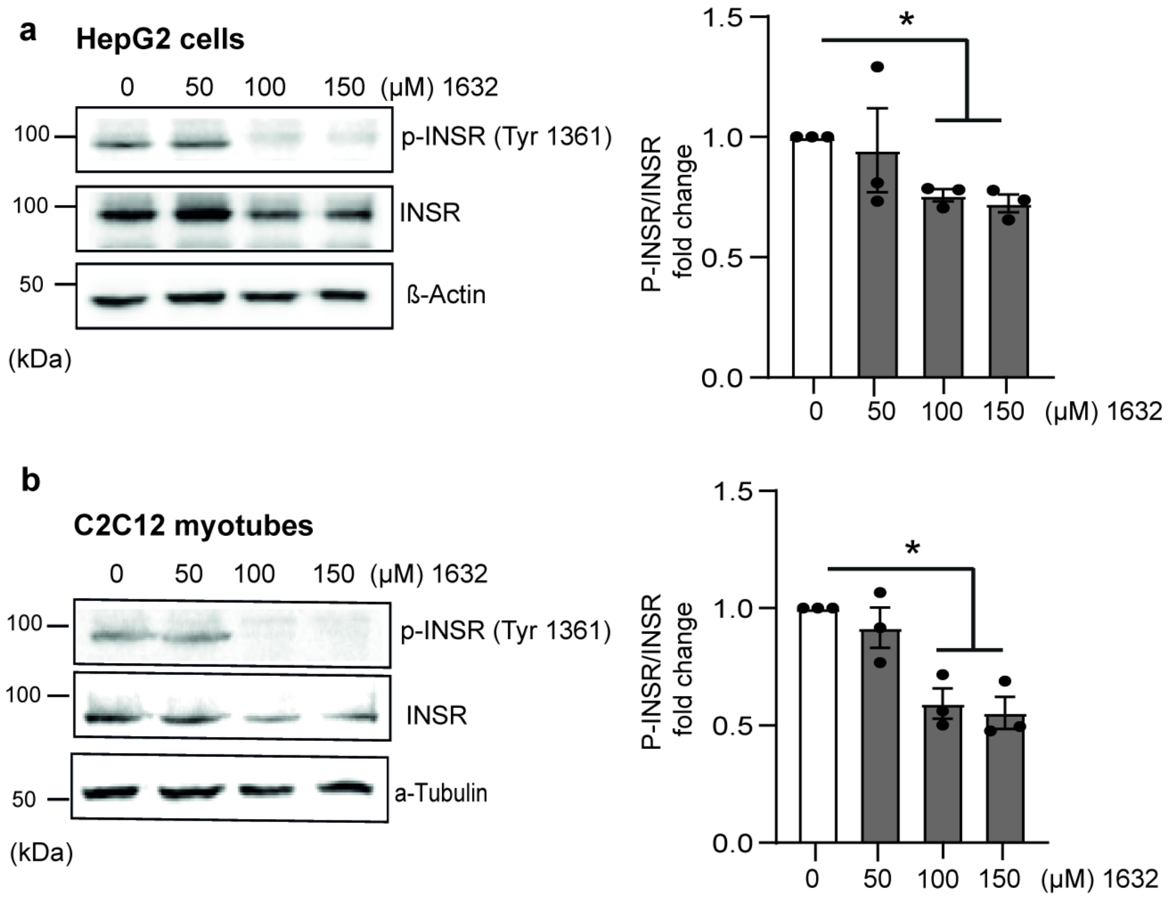

Supplementary Figure 3. 1632 decreases Insulin receptor tyrosine 1361 phosphorylation in cells.

a), b) Immunoblot and densitometric analyses for P-INSR (Tyr 1361) and INSR in HepG2 and C2C12 cells treated with 1632 after 4 or 6 days of treatment respectively (n=3 independent experiments, (HepG2), 0 vs 100 µM, P=0.0129; 0 vs 150 µM, P=0.0235; (C2C12), 0 vs 100 µM, P=0.0240; 0 vs 150 µM, P=0.0224). Values are mean ±SEM. *P < 0.05; **P < 0.01, ***P < 0.001. Groups were compared using two-tailed unpaired Student's t-test. Uncropped images are shown in **Supplementary Figure 21.**

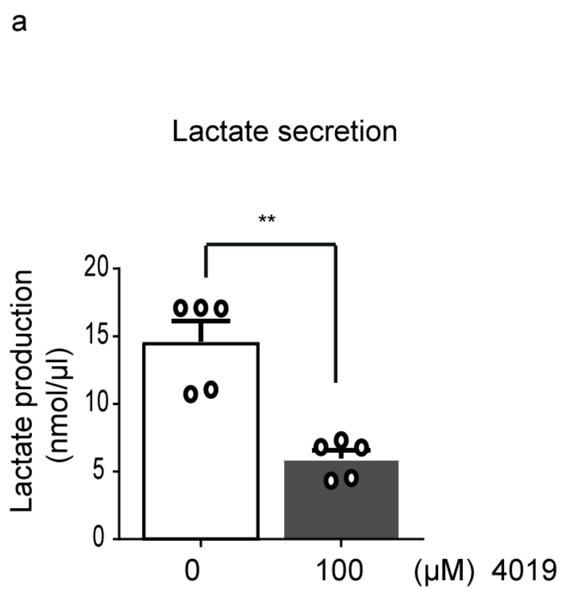

Supplementary Figure 4. Lin28B inhibition results in decreased lactate secretion in HepG2 cells.

a) Lactate concentration was measured in culture medium of HepG2 cells treated for 3 days with 4019 (n=5 biologically independent samples/group, P=0.0027). **b)** Lactate concentration was measured in culture medium of HepG2 cells transfected with siControl (siCtrl) or siLin28b. Lactate levels were normalized to total protein of the corresponding samples (n=3 biologically independent samples/ group, P=0.0042). Values are mean ±SEM. *P < 0.05; **P < 0.01, ***P < 0.001. Groups were compared using two-tailed unpaired Student's t-test.

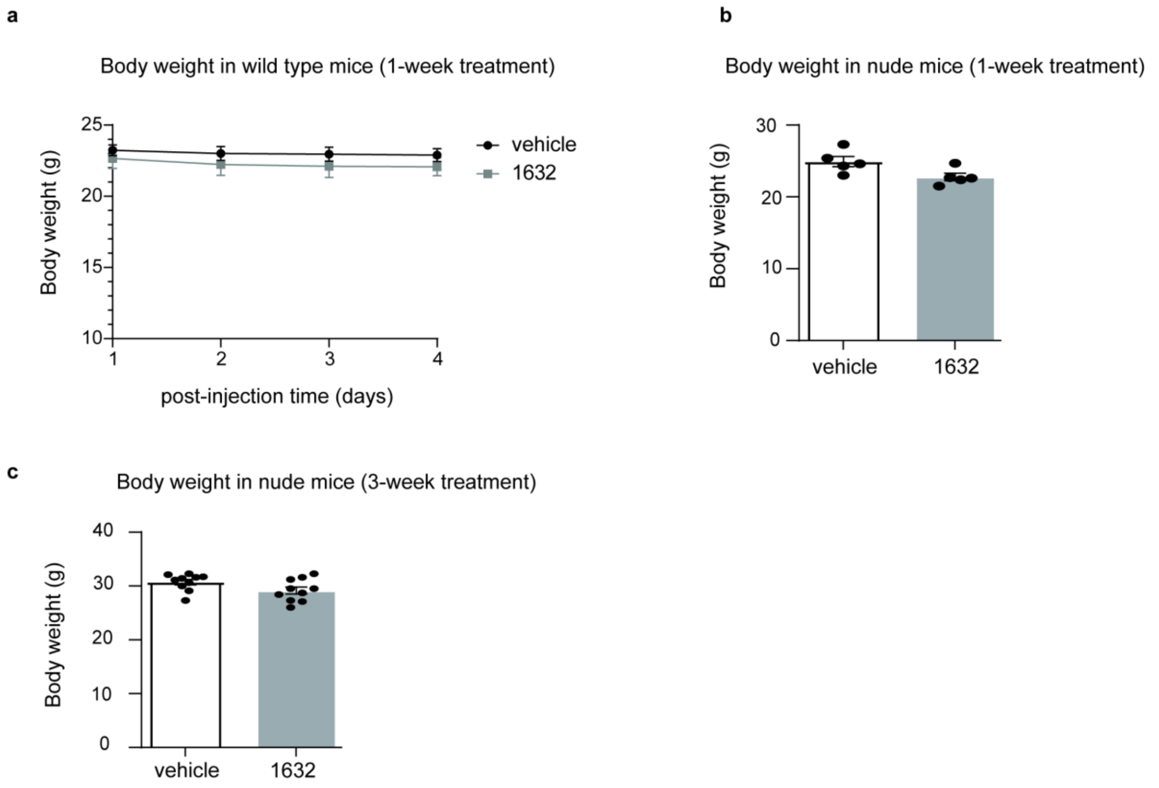

Supplementary Figure 5. 1632 treatment did not change body weight in mice.

a) Body weight during 5-day treatment in 8-week-old male wild type mice (n=8 mice/group)**. b)** Body weight at the end of 5-day treatment in 8-week-old male nude mice (n=5 mice/group). **c)** Body weight at the end of the 3-week treatment in 10-week-old male nude mice (n=10 mice/group). Values are mean ±SEM. Groups were compared using two-tailed unpaired Student's t-test.

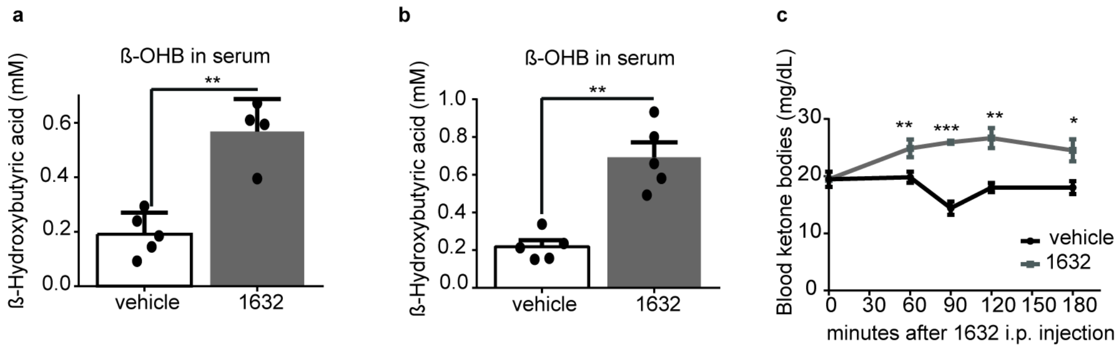

Supplementary Figure 6. 1632 treatment increases circulating ketone body levels in nude mice.

a) Serum β-OHB levels in 1632 and vehicle-treated 8-week-old male nude mice after daily IP injections (50 mg/kg) for 5 days (control n=5, 1632 n=4, P=0.0029). **b)** Serum β-OHB levels in 1632 and vehicle-treated 10-week-old, male nude mice after 3 weeks of treatment. 1632 or vehicle was administered every other day (n=5 mice/group, P=0.0020). **c)** Blood ketone body (β-OHB, acetone, acetoacetate) levels at the indicated time points post-1632 injection (n=5 mice/group; 60 min, P=0.0296; 90 min, P=0.0002; 120 min, P=0.0048; 180 min, P=0.0257). Values are mean ±SEM. *P < 0.05; **P < 0.01, ***P < 0.001. NS: not significant. Groups were compared using two-tailed unpaired Student's t-test.

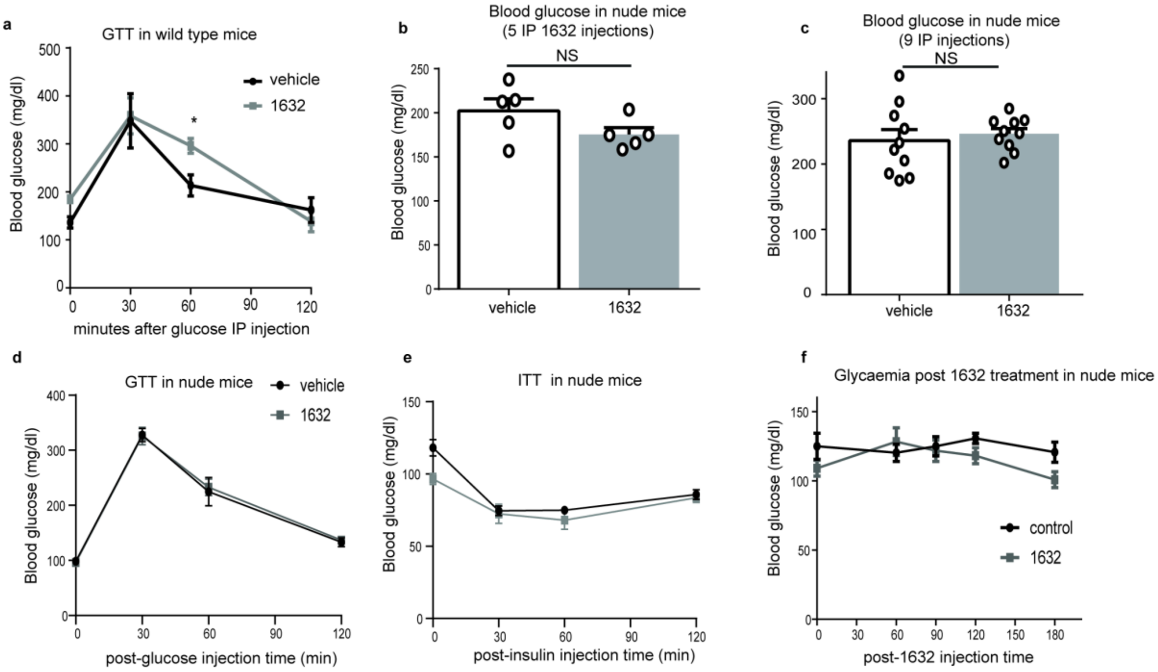

Supplementary Figure 7. 1632 Treatment caused moderate glucose intolerance in wild type mice but did not alter blood glucose levels in nude mice.

a) 7-week-old, male C57BL/6J wild-type mice received IP injections of 1632 or vehicle for 5 consecutive days, while mice were fed *ad libitum*. Mice were overnight fasted, followed by IP injection with glucose (2 mg/g body weight), and a glucose tolerance test was performed on the 5th day of treatment (n=4 mice/group, 60 min, P=0.0101). **b)** Blood glucose levels at the end of 5-day 1632 treatment in 8-week-old, male nude mice (n=5 mice/group). **c)** Blood glucose levels at the end of 3-week 1632 treatment in 10-week-old male nude mice (n=10 mice/group). **d)** Glucose tolerance test on the 13th day of treatment in male nude mice (n=5 mice/group). **e)** Insulin tolerance test on the 13th day of treatment in male nude mice (n=5 mice/group). **f)** Blood glucose levels at the indicated time points post-1632 injection (n=5 mice/group). Values are mean ±SEM. *P < 0.05; **P < 0.01, ***P < 0.001. Groups were compared using two-tailed unpaired Student's t-test.

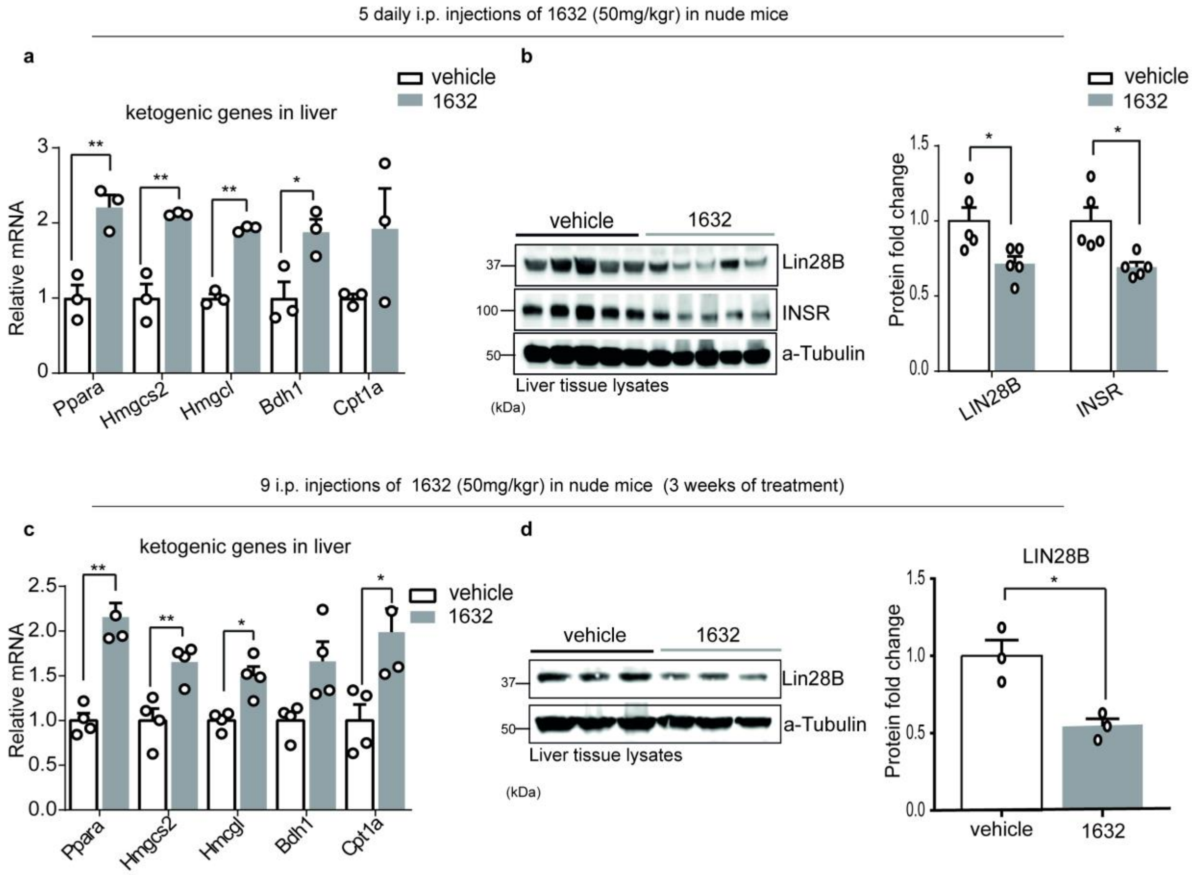

Supplementary Figure 8. 1632 treatment increased expression of ketogenic genes in livers of nude mice.

a) Expression of genes involved in ketogenesis in liver of 8-week-old male nude mice after 1 week of 1632 treatment**,** as determined using real-time PCR (n=3 mice/group; Ppara, P=0.0074; Hmgcs2, P=0.0265; Hmgcl, P=0.0010; Bdh1, P=0.0363). β-Actin was used as a housekeeping gene. **b)** Representative Immunoblot analysis and relative bar graph quantification for Lin28B and INSR in liver lysates from 1632 and vehicle-treated 8-week-old male nude mice after 1 week of treatment (n=5 mice/group; Lin28B, P=0.0309; INSR, P=0.0246). a-Tubulin was used as a loading control. **c)** Expression of genes involved in ketogenesis in liver of 10-week-old male nude mice after 3 weeks of 1632 treatment, as determined using real-time PCR (n=4 mice/group; Ppara, P=0.0017; Hmgcs2, P=0.0094; Hmgcl, P=0.0131; Cpt1a, P=0.0243). β-Actin was used as a housekeeping gene. **d)** Representative immunoblot analysis and relative bar graph quantification for Lin28B in liver lysates from 1632 and vehicle-treated 10-week-old male nude mice after 3 weeks of treatment (n=3 mice/group; P=0.0291). a-Tubulin was used as a loading control. Values are mean ±SEM. *P < 0.05; **P < 0.01; ***P < 0.001. Groups were compared using two-tailed unpaired Student's t-test. Uncropped images are shown in **Supplementary Figure 22.**

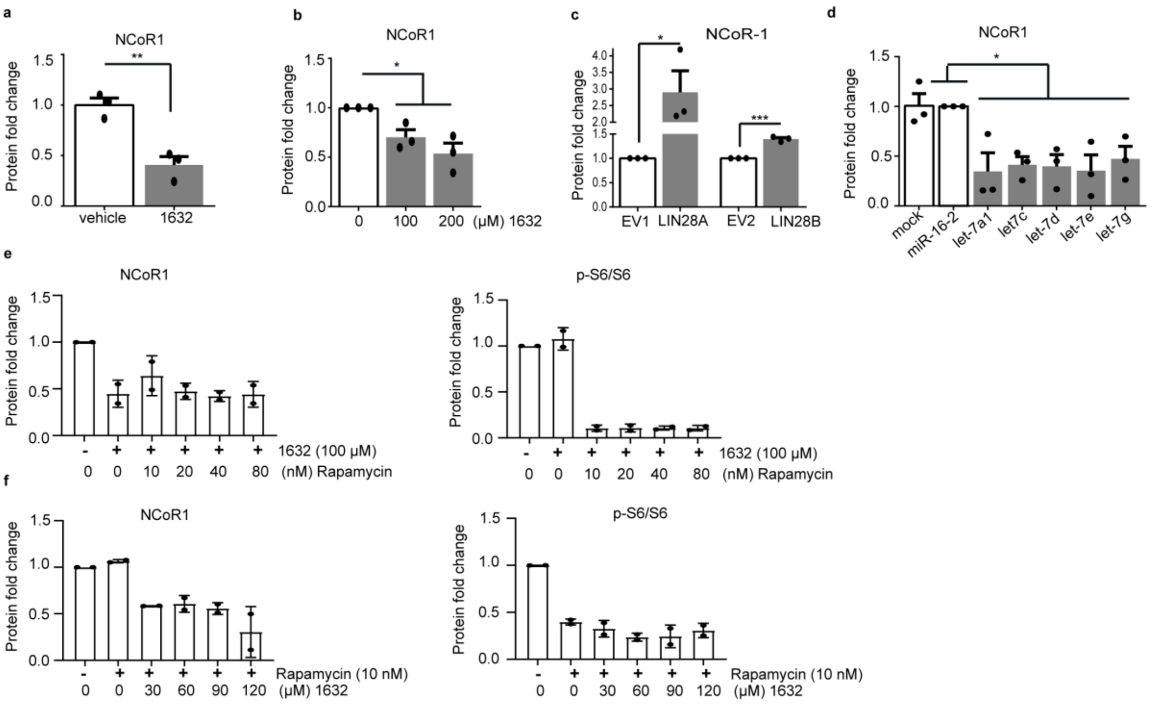

Supplementary Figure 9. Densitometric analyses for immunoblots in Fig.3

a)-f) Protein quantification for immunoblots in **Figs. 3d-g** (n=3 independent experiments; P=0.0059 (a); 0 vs 100 μM, P=0.0168; 0 vs 200 μM, P=0.0125; EV1 vs Lin28A, P=0.0424; EV2 vs Lin28B, P=0.0002; mock vs let-7a1, P=0.0426; mock vs let-7c, P=0.0159; mock vs let-7d, P=0.0238; mock vs let-7e, P=0.0315; mock vs let-7g, P=0.0390) and **Figs. 3k, 3m** (n=2 independent experiments). Immunoblot analyses in **Fig. 3k**, **m** were performed twice with comparable results. Values are mean ±SEM. *P < 0.05; **P < 0.01; ***P < 0.001. Groups were compared using two-tailed unpaired Student's t-test.

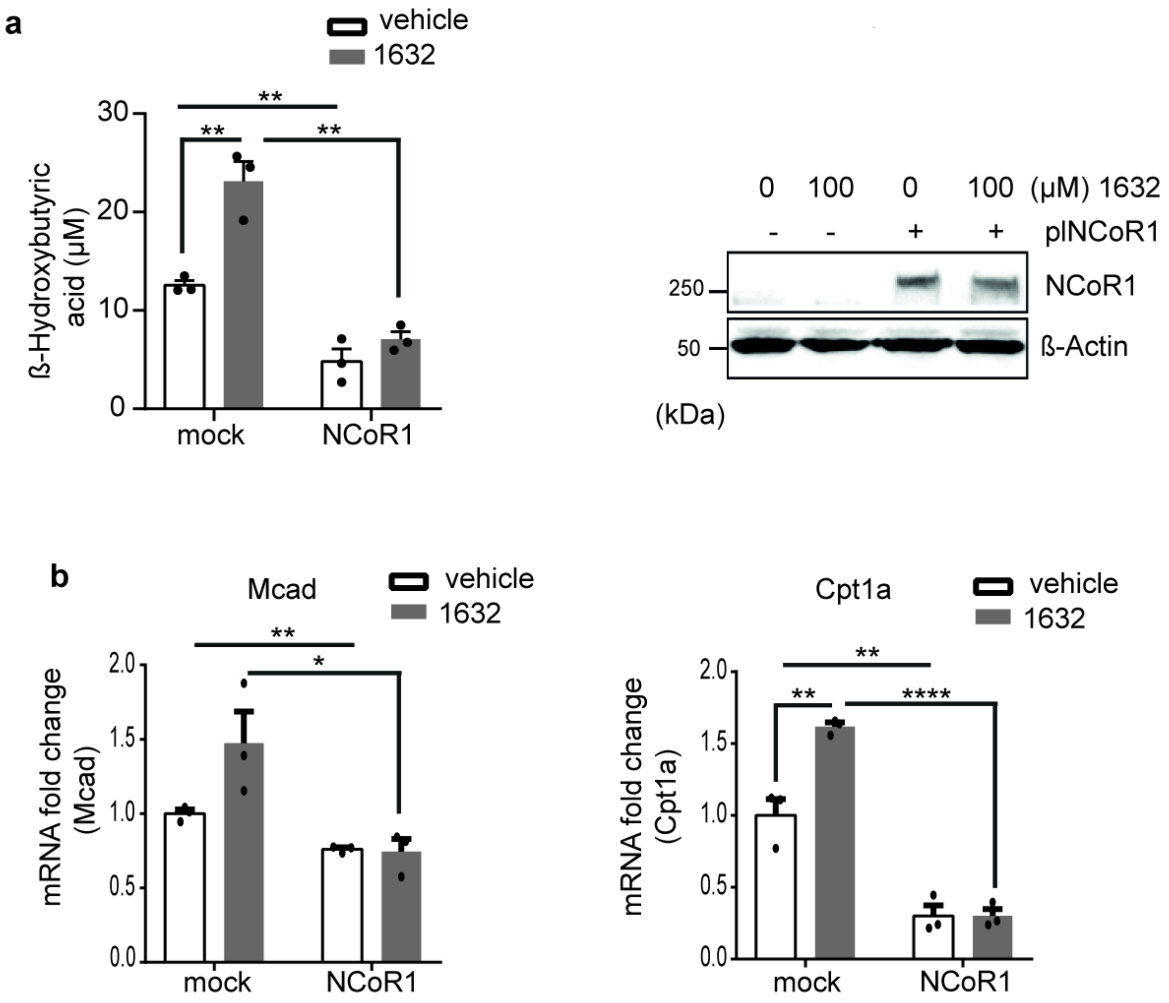

Supplementary Figure 10. NCoR1 overexpression abrogates the 1632-mediated ketogenesis activation.

a) β-OHB levels were measured in the growth medium of HepG2 cells treated with vehicle or 1632 (100 μM) for 72 h with or without NCoR1 overexpression in the presence of a Fatty Acid (F.A.) mixture of oleate and palmitate (n=3 biologically independent samples/group; mock vehicle vs mock 1632, P=0.0068; mock vehicle vs NCoR1 vehicle, P=0.0046; mock 1632 vs NCoR1 1632, P=0.0017). β-OHB levels were normalized to the total protein of the corresponding samples. NCoR1 overexpression was confirmed by immunoblot. **b)** Relative mRNA levels of the PPARa target genes Mcad and Cpt1a in HepG2 cells treated as described in **a** (n=3 biologically independent samples/group; Mcad, mock vehicle vs NCoR1 vehicle, P=0.0016; vehicle 1632 vs NCoR1 1632, P=0.0335; Cpt1a, mock vehicle vs mock 1632, P=0.0065; mock vehicle vs mock NCoR1, P=0.0067; mock 1632 vs NCoR1 1632, P<0.0001)**.** β-Actin served as a housekeeping gene. Values are mean ±SEM. *P < 0.05; **P < 0.01, ***P < 0.001, ****P < 0.0001. NS: not significant. Groups were compared using two-tailed unpaired Student's t-test. Uncropped images are shown in **Supplementary Figure 23.**

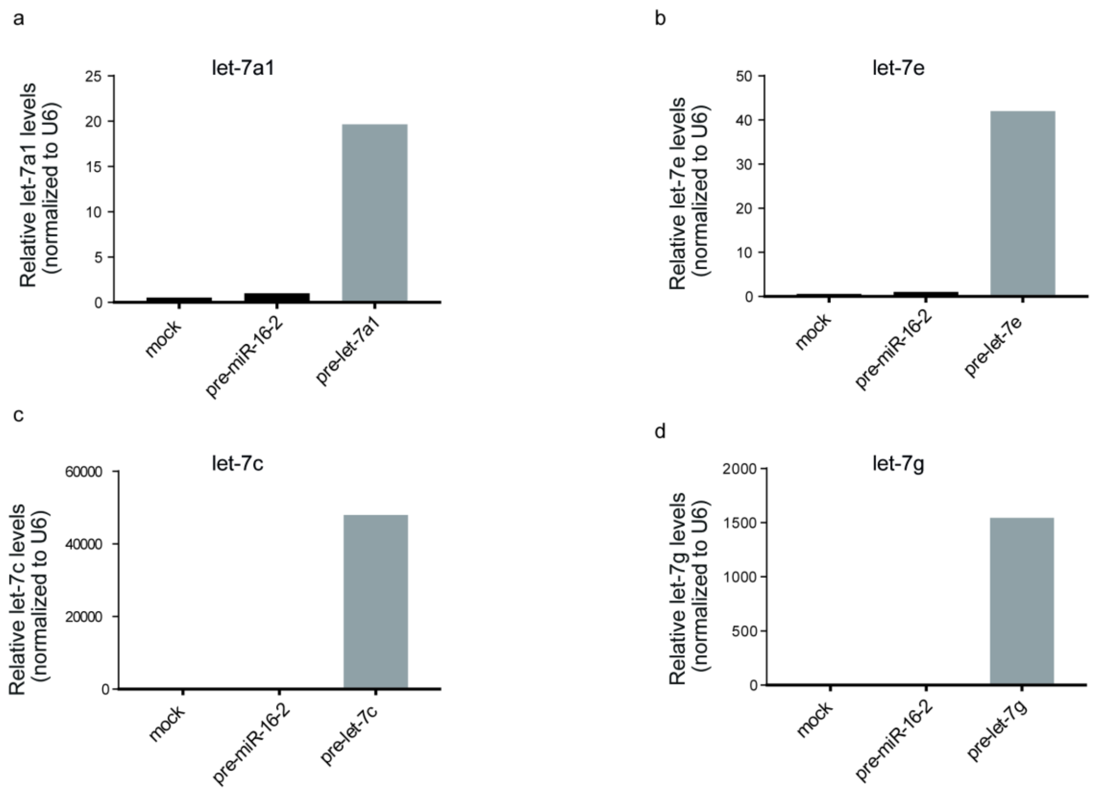

Supplementary Figure 11. Let-7 overexpression validation in let-7-transfected HepG2 cells.

Taqman PCR for: **a)** let-7a1 **b)** let-7e **c)** let-7c and **d)** let-7g levels in HepG2 cells 48 h post-transfection with mock, pre-miR16-2, and pre-let-7a1, pre-let-7e, pre-let-7c, pre-let-7g, respectively. U6 snRNA served as internal control.

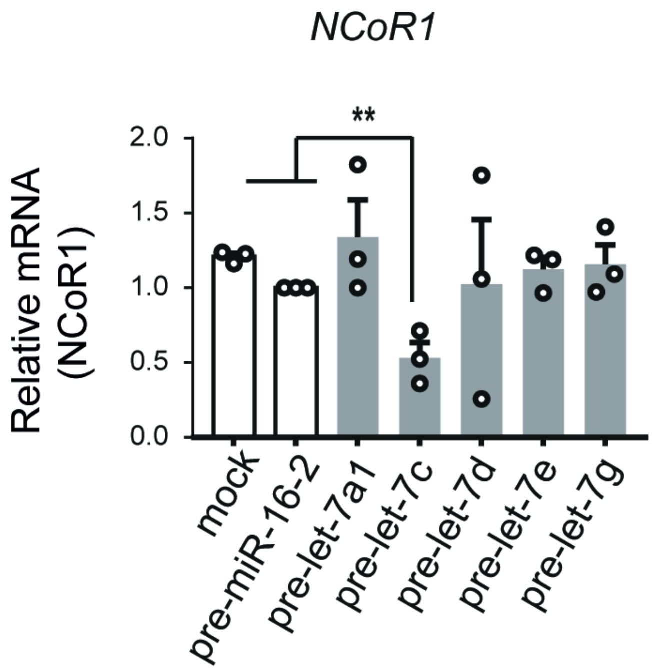

Supplementary Figure 12. Let-7c represses levels of NCoR1 mRNA.

NCoR1 mRNA levels in HepG2 cells transfected with pre-miR-16-2, pre-let-7a1, pre-let-7c, pre-let-7d, pre-let-7e and pre-let-7g (n=3 biologically independent samples/group; P=0.0029). β-Actin was used as a housekeeping gene. Values are mean ±SEM. *P < 0.05; **P < 0.01; ***P < 0.001. Groups were compared using two-tailed unpaired Student's t-test.

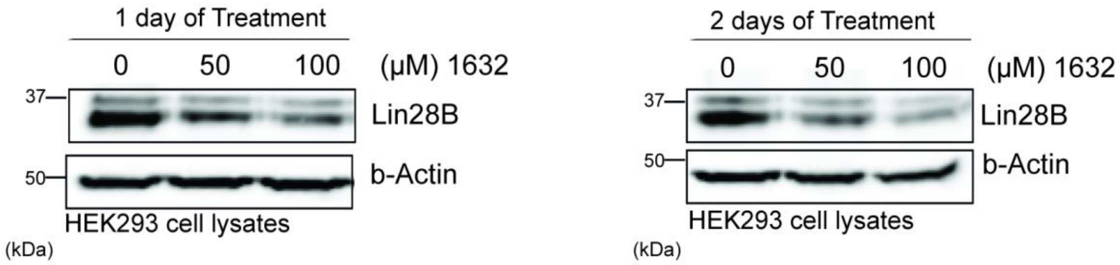

Supplementary Figure 13. 1632 treatment inhibits Lin28B in HEK293T cells.

Immunoblot analysis for Lin28B in HEK293T cells after one or two days of 1632 treatment in HEK293T cells. β-Actin expression was used as a loading control. The experiment was repeated three times independently with similar results. Uncropped images are shown in **Supplementary Figure 24.**

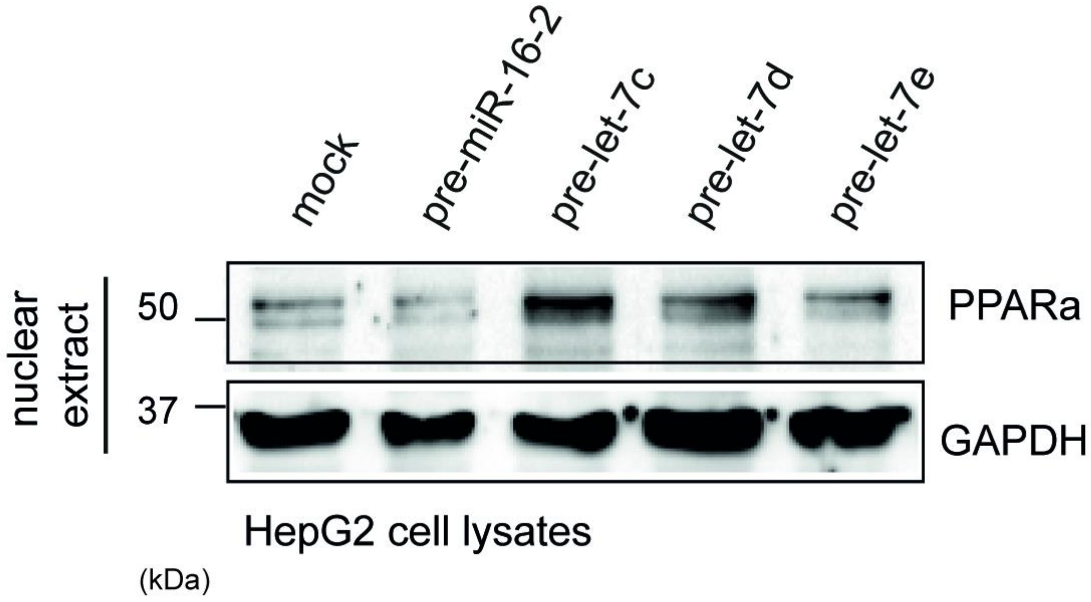

Supplementary Figure 14. Transfection of HepG2 cells with let-7 induces expression of PPARα.

Immunoblot analysis for PPARα in nuclear extracts from HepG2 cells after 48 h of transfection with pre-miR-16-2, pre-let7c, pre-let7d and pre-let7e. GAPDH expression was used as a loading control. The experiment was repeated three times independently with similar results. Uncropped images are shown in **Supplementary Figure 25.**

a b

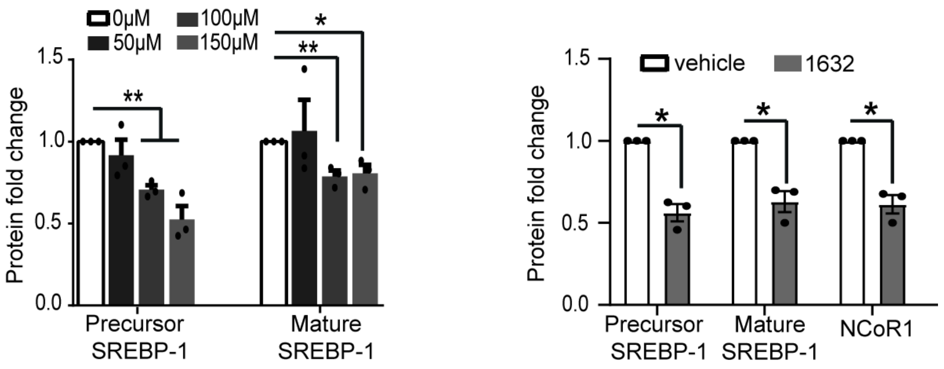

Supplementary Figure 15. Densitometric analyses for immunoblots in Figure 4.

a), b) Protein quantification for immunoblots in **Figs. 4e, 4h** (n=3 independent experiments; (a), P-SREBP1, 0 vs 100 μM, P=0.0088; P-SREBP1, 0 vs 150 μM, P=0.0282; M-SREBP1, 0 vs 100 μM, P=0.0277; M-SREBP1, 0 vs 150 μM, P=0.0209; (b) P-SREBP1, P=0.0141; M-SREBP1, P=0.0290; NCoR1, P=0.0207). Values are mean ±SEM. *P < 0.05; **P < 0.01; ***P < 0.001. Groups were compared using two-tailed unpaired Student's t-test.

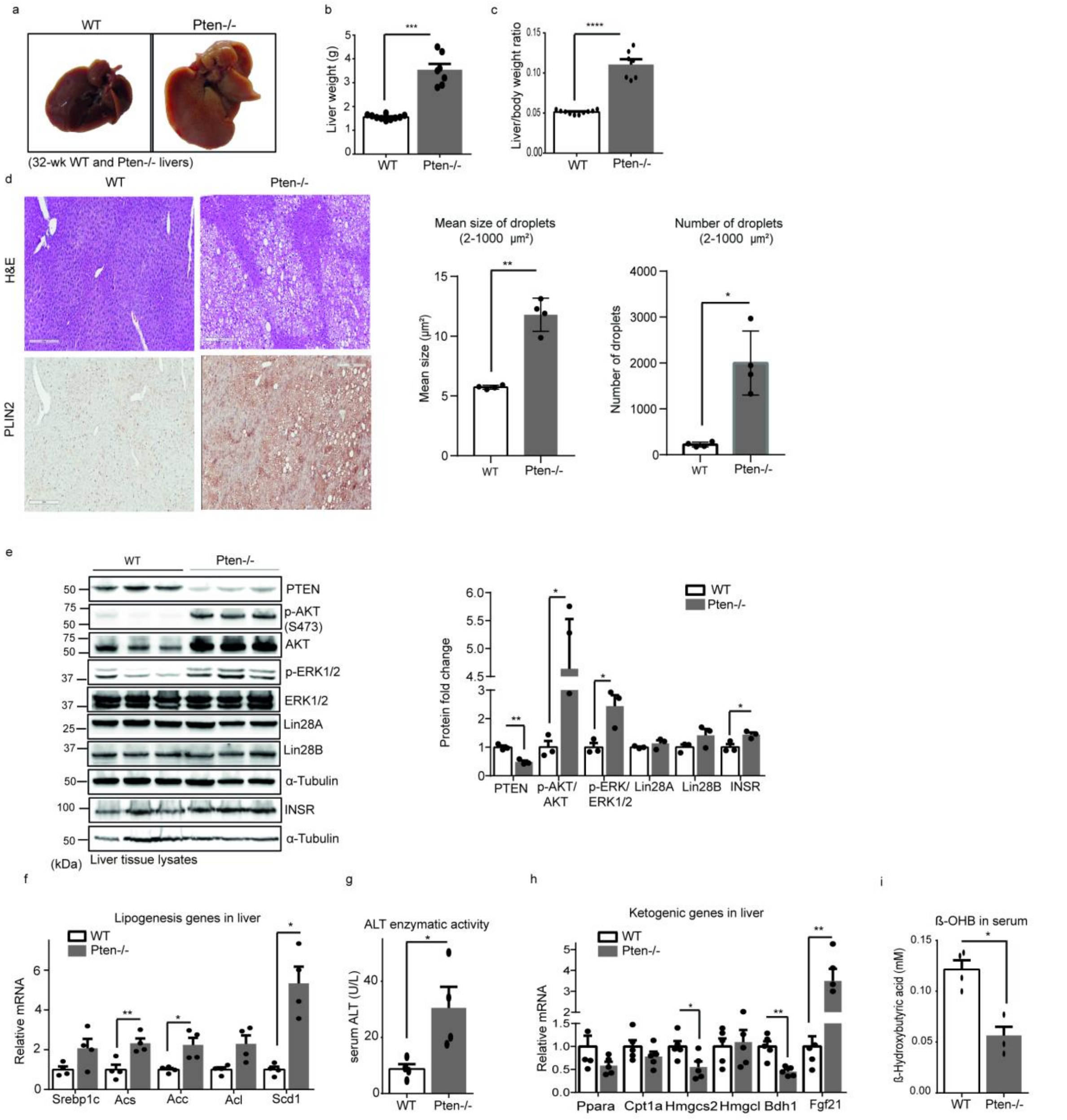

Supplementary Figure 16. Liver-specific Pten$^{-/-}$ male mice develop fatty liver phenotype.

a) Representative images of livers from 32-week-old male wild type and Pten$^{-/-}$ mice. **b), c)** Liver weight and liver to body weight ratio (7-10 mice/group; P=0.0002; P<0.0001). **d)** H&E and PLIN2 staining of liver sections from 32-wk-old wild type and Pten$^{-/-}$ mice. Scale bars correspond to 300 μM. Lipid droplet size and number were quantified by Image J (n=4 histological images/group; P=0.0029; P=0.0143). **e)** Immunoblot analysis and relative bar graph quantification for PTEN, P-AKT, AKT, P-ERK1/2, ERK1/2 Lin28A, Lin28B and INSR in liver lysates from wild type and Pten$^{-/-}$ mice at 32 weeks (n=3 mice/group; PTEN, P=0.0029; P-AKT/AKT, P=0.0478; P-ERK/ERK, P=0.0256; INSR, P=0.0287). a-Tubulin served as a loading control. **f)** Relative mRNA levels of ipogenic genes in wild type and Pten$^{-/-}$ livers at 32 weeks (n=4 mice/group; Acs, P=0.0092; Acc, P=0.0390; Scd1, P=0.0125). β-Actin was used as a housekeeping gene. **g)** Serum ALT activity of wild type and Pten$^{-/-}$ male mice at 32 weeks (n=4 mice/group; 0.0303). **h)** Relative mRNA levels of ketogenic genes in wild type and Pten$^{-/-}$ livers at 32 weeks (n=5 mice/group; Hmgcs2, P=0.0293; Bdh1, P=0.0055; Fgf21, P=0.0097). β-Actin was used as a housekeeping gene. **i)** Serum β-OHB levels in WT and Pten$^{-/-}$mice at 32 weeks (n=4 mice/group; P=0.0020). Uncropped images for (**e**) are shown in **Supplementary Fig.26.** Values are mean ±SEM.

*P < 0.05; **P < 0.01; ***P < 0.001, ****P < 0.0001. Groups were compared using two-tailed unpaired Student's t-test.

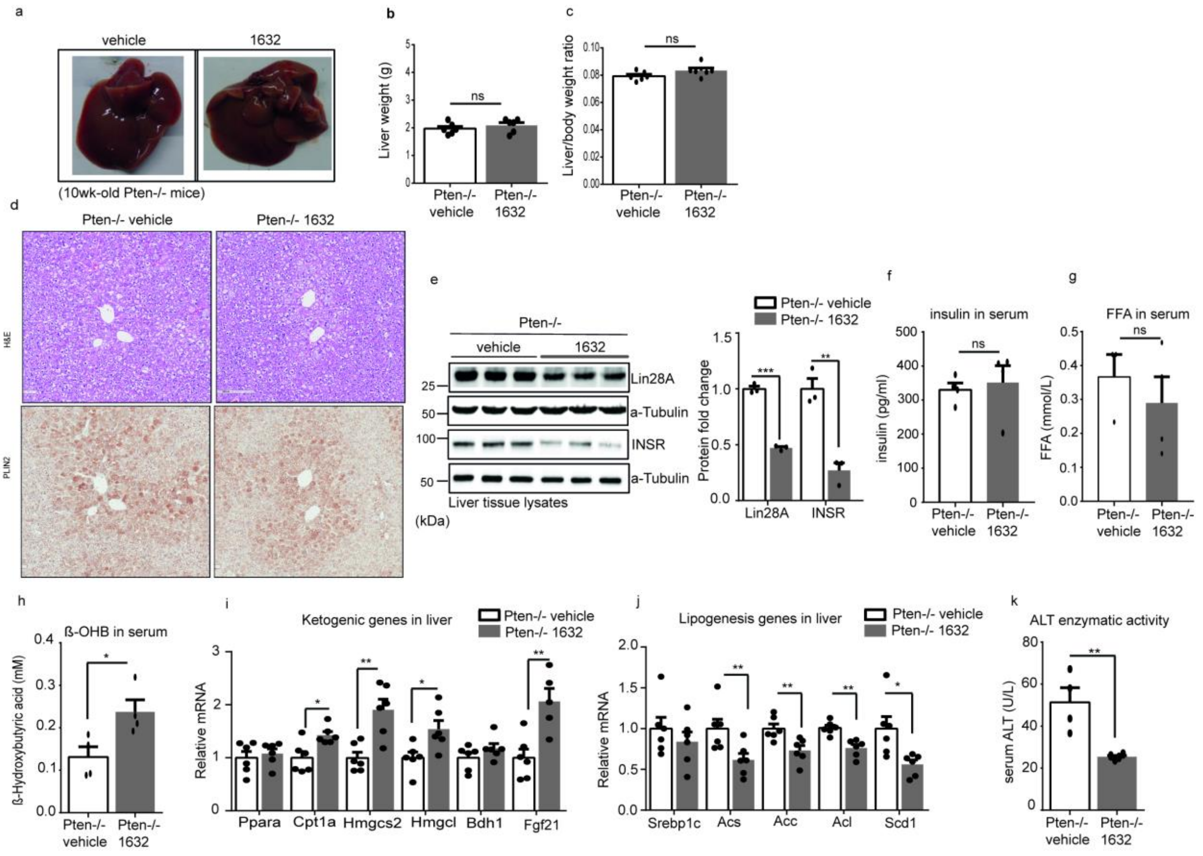

Supplementary Figure 17. C1632 Treatment in 10-wk old liver-specific Pten$^{-/-}$ male mice.

a) Representative liver images of 10-week-old vehicle or 1632-treated Pten$^{-/-}$ male mice after 4 week of treatment (3 IP injections/week at a dose of 50 mg/kg). **b) c)** Liver weight and liver to body weight ratio (n=6 mice/group) **d)** H&E and PLIN2 staining of liver sections from 10-wk-old vehicle and 1632 Pten$^{-/-}$ mice. Scale bars correspond to 300 μM. **e)** Immunoblot analysis and relative bar graph quantification for LIN28A, INSR in liver lysates from 10-week-old vehicle and 1632-treated Pten$^{-/-}$ mice. (n=3 mice/group; Lin28A, P=0.0003; INSR, P=0.0043). a-Tubulin served as a loading control. **f-h)** Serum insulin, free fatty acids (FFA) and β-OHB levels in Pten$^{-/-}$ mice treated with vehicle or 1632 (n=3-4 mice/group; P=0.0294). **i), j)** Relative mRNA levels of ketogenic and lipogenic genes in livers from 10-week-old vehicle and 1632-treated Pten$^{-/-}$ mice (n=5-6 mice/group; Cpt1a, P=0.0140; Hmgcs2, P=0.0041; Hmgcl, P=0.0211; Fgf21, P=0.0022; Acs, P=0.0236; Acc, P=0.0095; Acl, P=0.0012; Scd1, P=0.0293). β-Actin was used as a housekeeping gene. **k)** Serum ALT activity of Pten$^{-/-}$ mice treated with vehicle or 1632 (n=4 mice/group; P=0.0322). Uncropped images for (**e**) are shown in **Supplementary Fig.26**. Values are mean ±SEM. *P < 0.05; **P < 0.01; ***P < 0.001. Groups were compared using two-tailed unpaired Student's t-test.

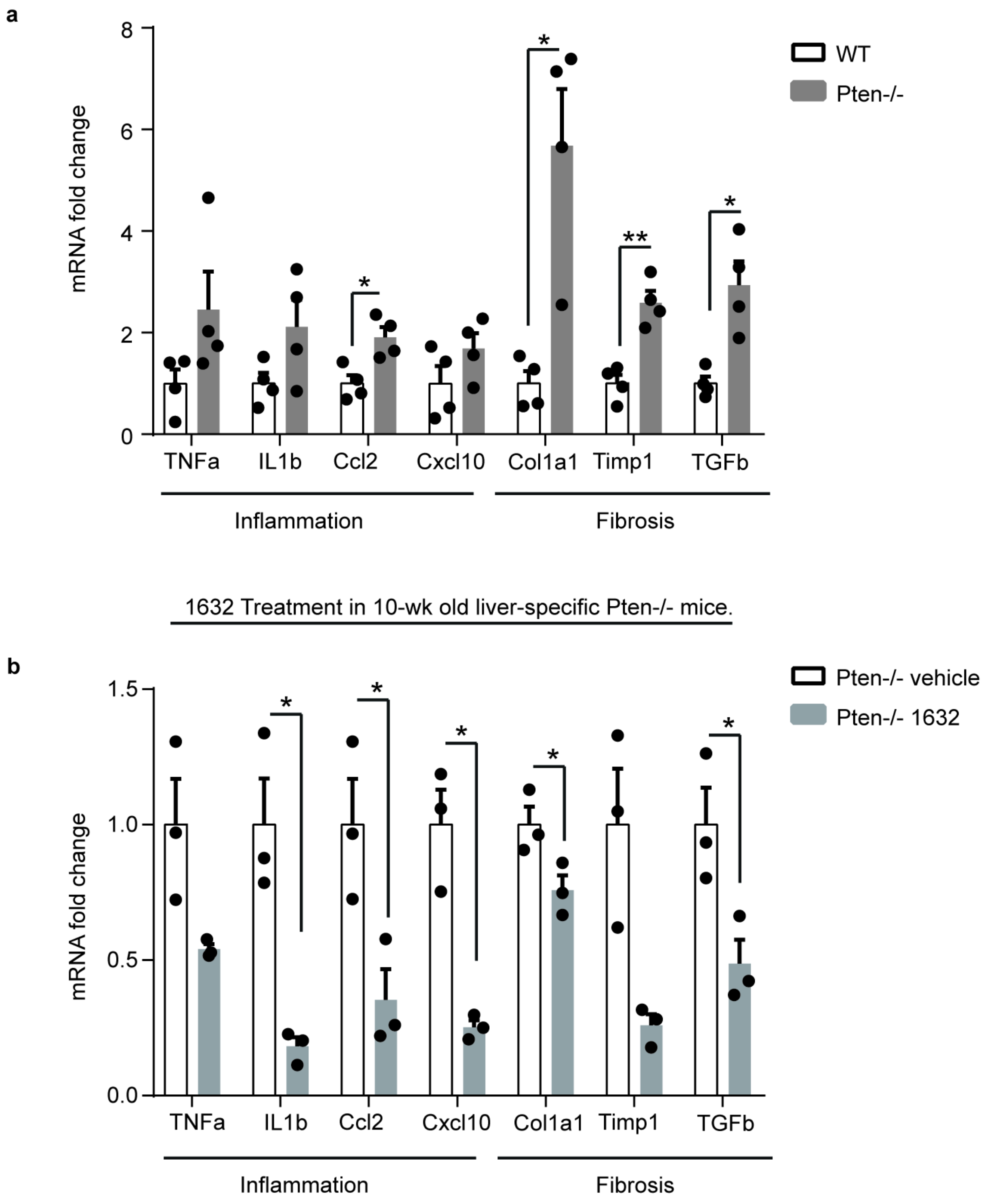

Supplementary Figure 18. 1632 decreases inflammation and fibrosis markers in Pten-deficient livers.

Expression of genes involved in inflammation and fibrosis in livers of **(a)** 32-week-old wild type and Pten-/- mice (n=4 mice/group), **b)** 10-week-old vehicle or 1632-treated Pten-/- mice after 4 weeks of treatment (3 IP injections/week at a dose of 50 mg/kg) (n=3 mice/group; (a), Ccl2, P=0.0133; Col1a1, P=0.0218; Timp1, P=0.0019; TGFb, P=0.0208; (b) IL1b, P=0.0363; Ccl2, P=0.0404; Cxcl10, P=0.0246; Col1a1, P=0.0498; TGFb, P=0.0427). *P < 0.05; **P < 0.01, ***P < 0.001. NS: not significant. Values are mean ±SEM. Groups were compared using two-tailed unpaired Student's t-test.

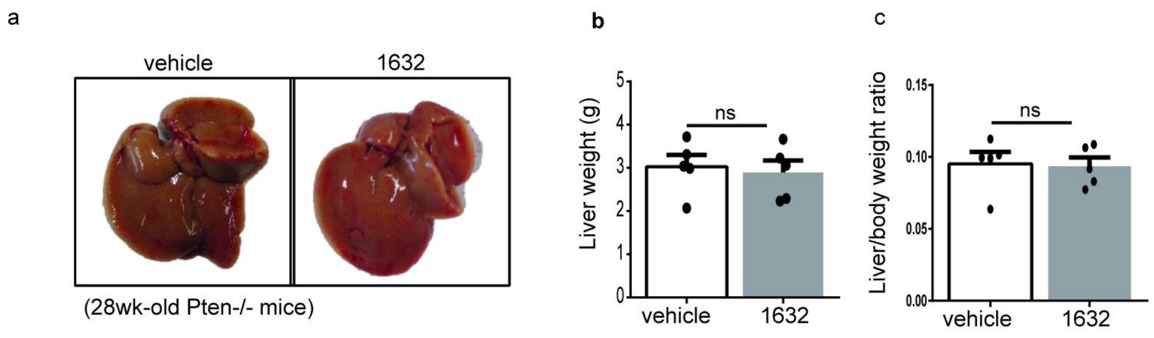

Supplementary Figure 19. 1632 Treatment does not alter liver/body weight in 28-wk old Pten$^{-/-}$ mice.

a) Representative images of livers from 28-week-old vehicle and 1632-treated Pten$^{-/-}$ male mice after 6 weeks of treatment (3 IP injections/week at a dose of 50 mg/kg). **b), c)** Liver weight and liver to body weight ratio of vehicle- and 1632-treated Pten$^{-/-}$ mice (n=5 mice/group). Values are mean ±SEM. Groups were compared using two-tailed unpaired Student's t-test.

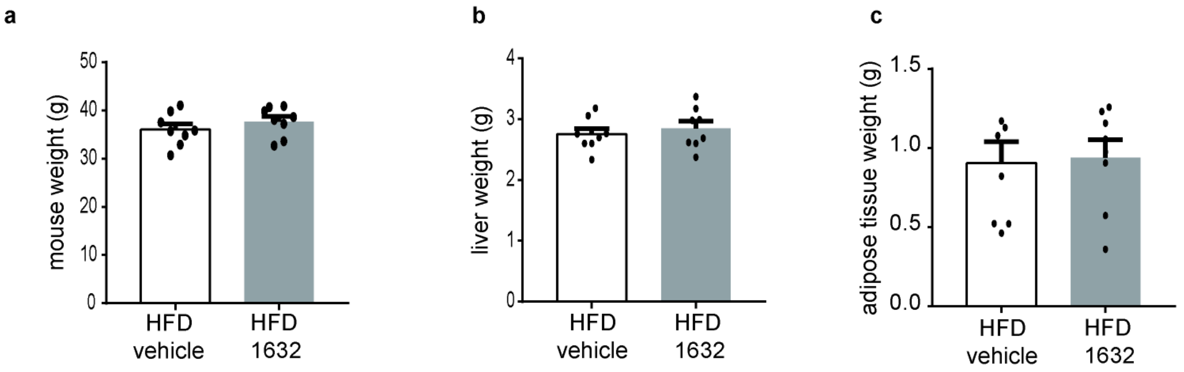

Supplementary Figure 20. Mouse, liver, and adipose tissue weights are not significantly different between vehicle- and 1632-treated mice on HFD.

a), b), c) Mouse, liver, and adipose tissue weights of vehicle- and 1632 treated mice fed with HFD (n=8 mice/group). Values are mean ±SEM. Groups were compared using two-tailed unpaired Student's t-test.

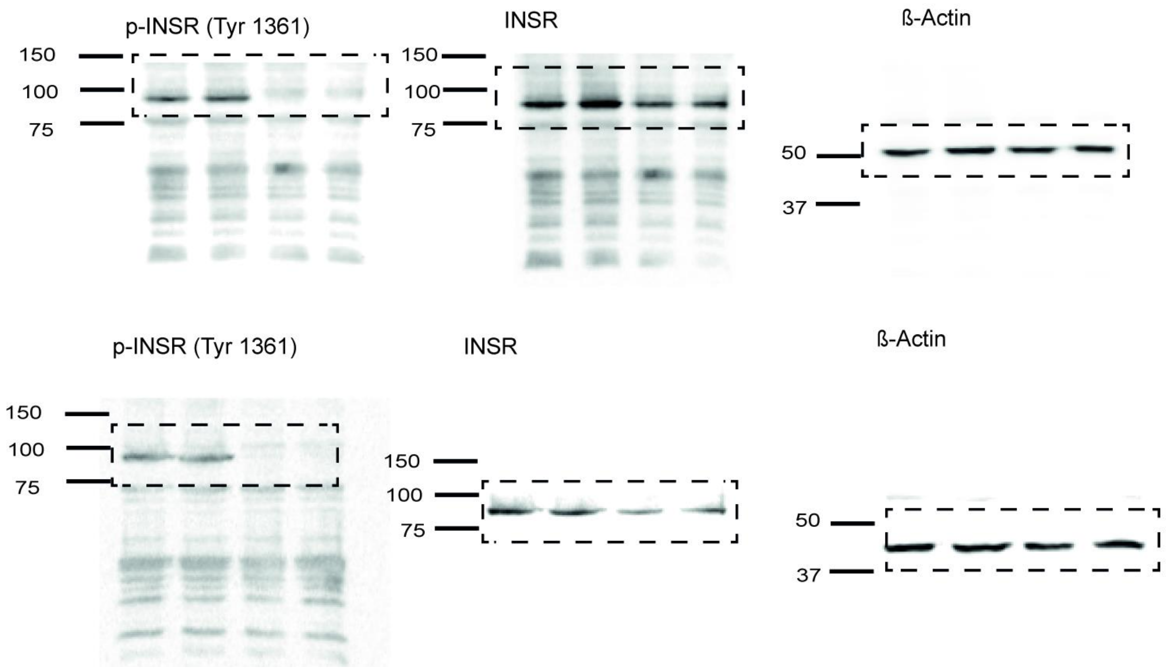

Supplementary Figure 21. Full blots for Supplementary Figure.3

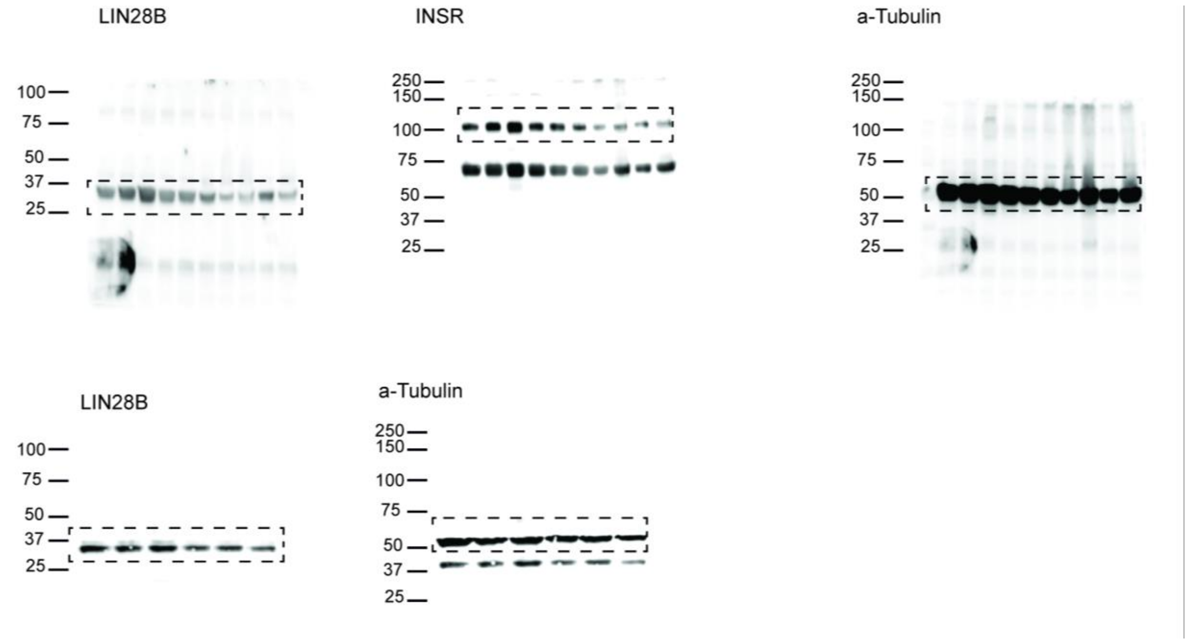

Supplementary Figure 22. Full blots for Supplementary Fig. 8b,d

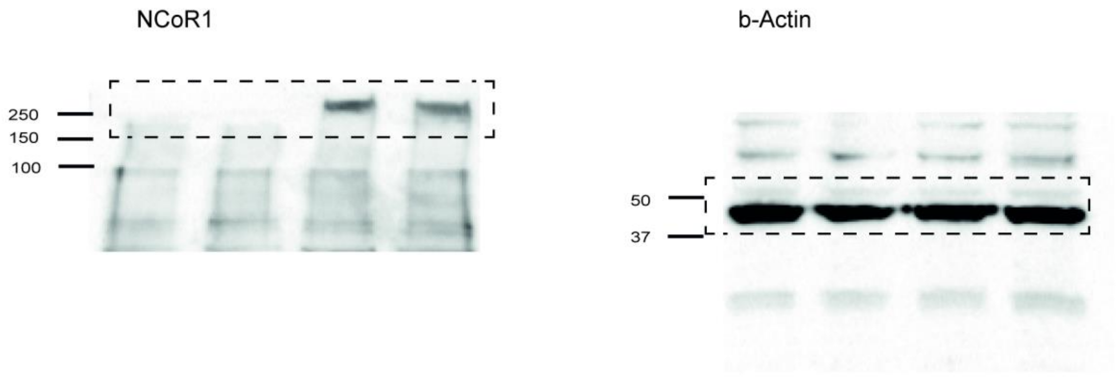

Supplementary Figure 23. Full blots for Supplementary Figure 10

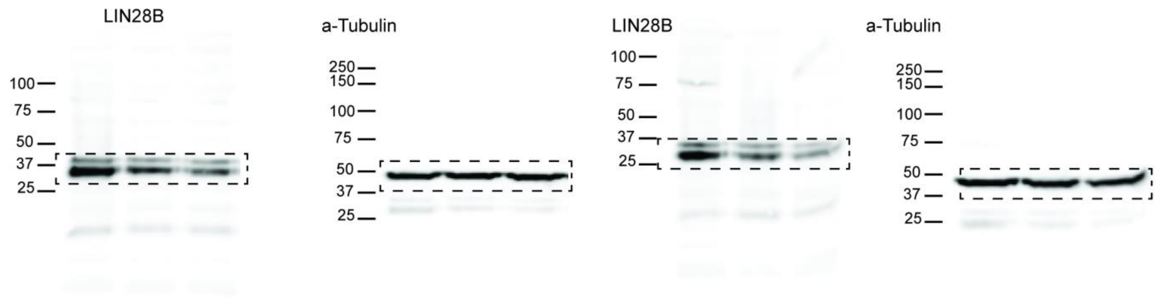

Supplementary Figure 24. Full blots for Supplementary Figure 13.

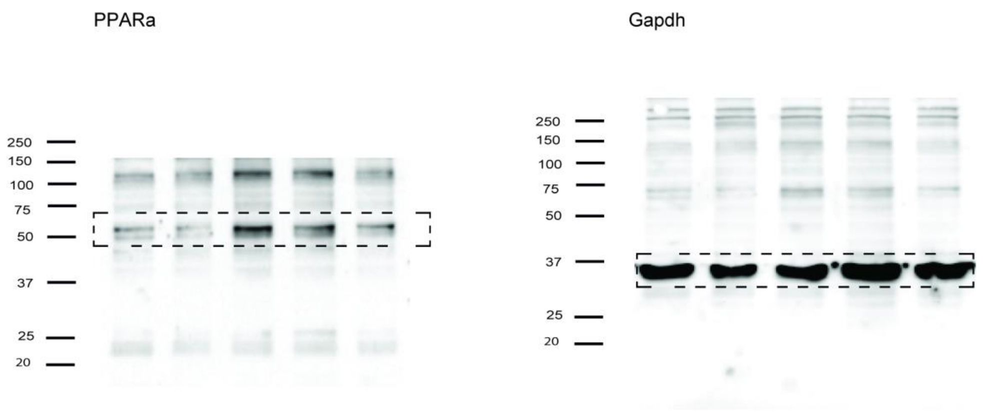

Supplementary Figure 25. Full blots for Supplementary Figure 14.

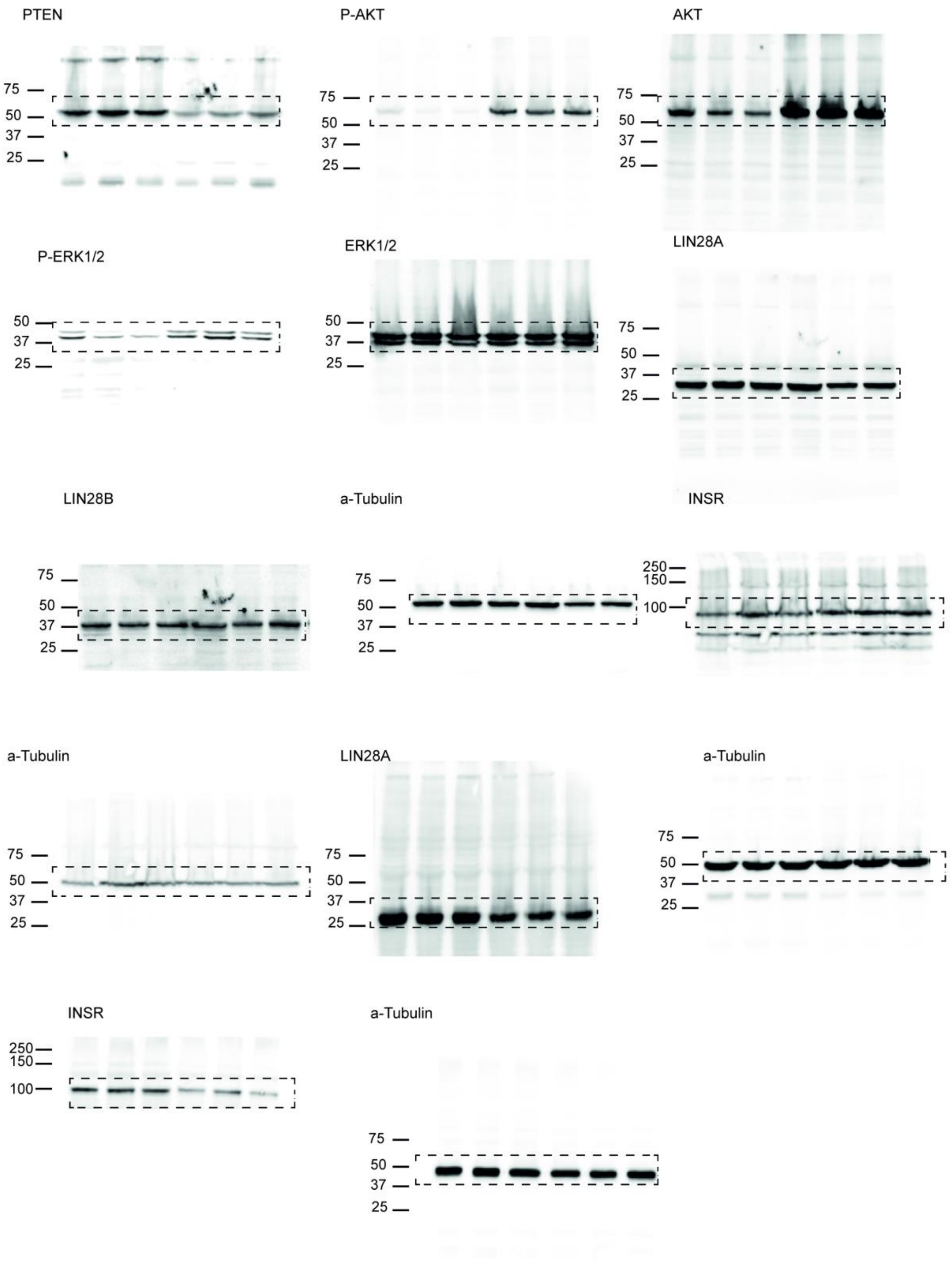

Supplementary Figure 26. Full blots for Supplementary Figures 16e and 17e

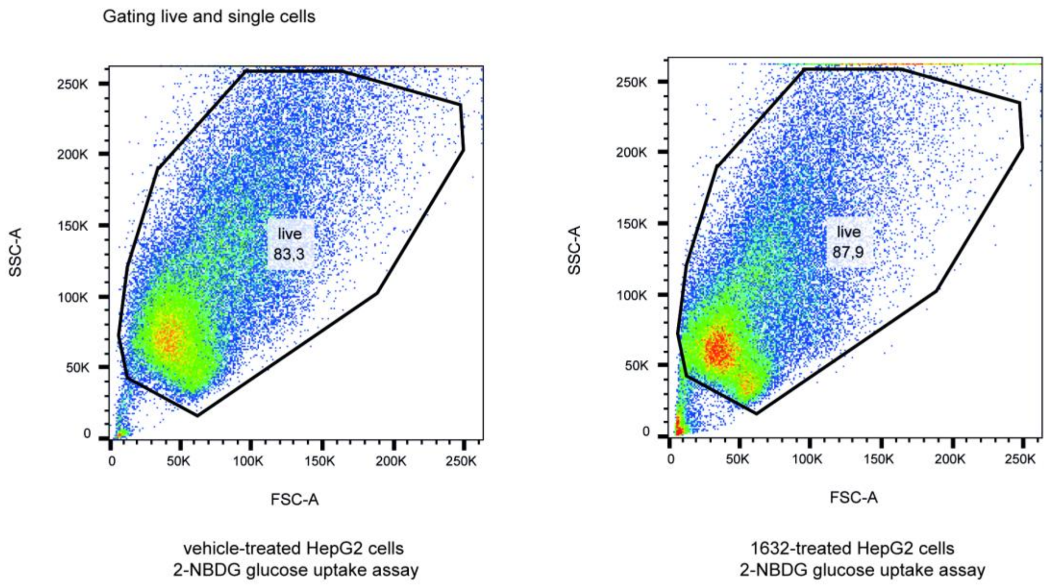

Supplementary Figure 27. The gating strategy for 2-NBDG glucose uptake assay.
Dead cells and cell doublets were excluded, and 2-NBDG fluorescence intensity was calculated from single, live vehicle- or 1632-treated HepG2 cells as shown in Fig.1h.

Pre-let-7-a1 UGAGGUAGUAGGUUGUAUAGUUUUAGGGUCACACCCACCACUGGGAGAUAACUAUACAAUCUACUGUCUUUC

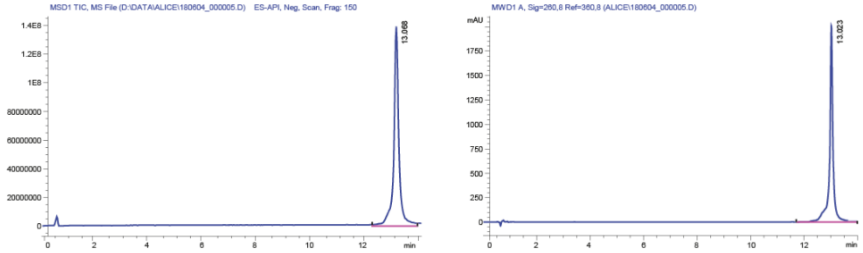

Deconvolution of Spectrum # 1 @ 12.920 - 13.269 min

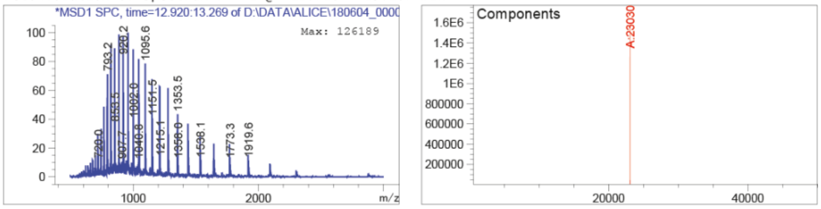

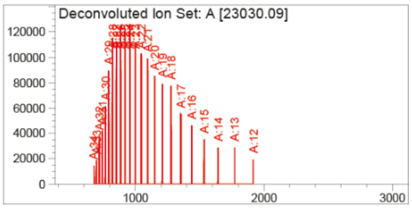

Component	Molecular Weight	Absolute Abundance	Relative Abundance
A	23030.09	1584280	100.00

*** End of Report ***

Calculated MS: 23030

Pre-let-7-c GAGGUAGUAGUUUGUACAGUUUGAGGGUCUAUGAUACCACCCGGUACAGGAGAUAACUGUACAGGCCACUGCCUUGC

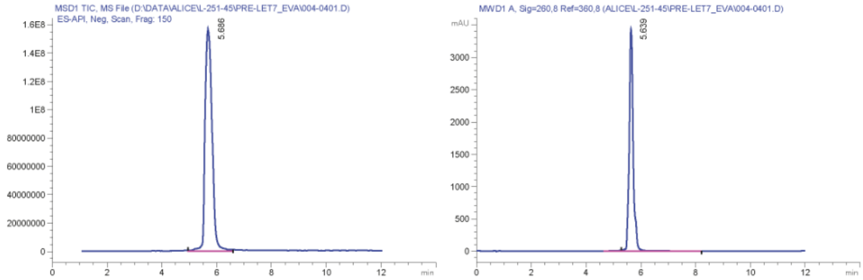

Deconvolution of Spectrum # 1 @ 5.683 min

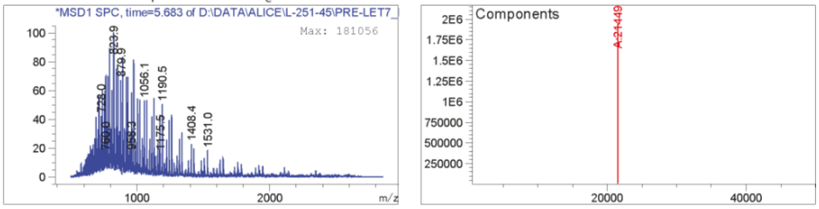

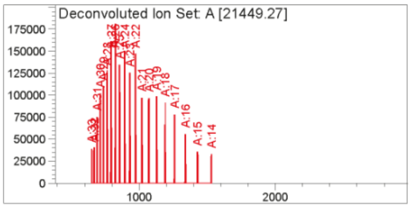

Component	Molecular Weight	Absolute Abundance	Relative Abundance
A	21449.27	1937607	100.00

*** End of Report ***

Calculated MS: 21449

Pre-let-7-d AGAGGUAGUAGGUUGCAUAGUUUUAGGGCAGGGAUUUUGCCCACAAGGAGGUAACUAUACGACCUGCUGCCUUUC

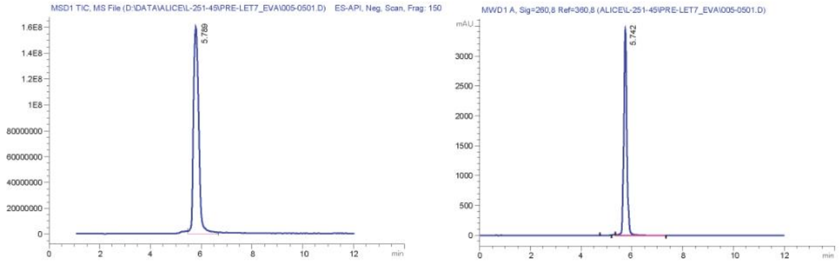

Deconvolution of Spectrum # 1 @ 5.857 - 5.882 min

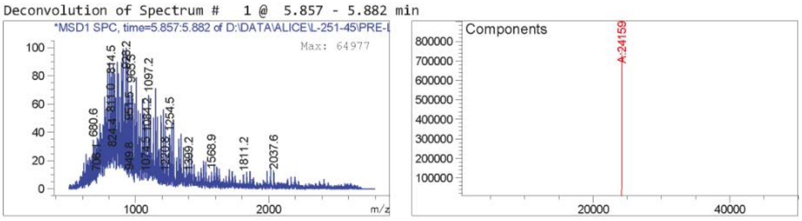

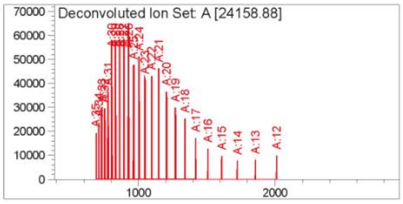

Component	Molecular Weight	Absolute Abundance	Relative Abundance
A	24158.88	809491	100.00

*** End of Report ***

Calculated MS: 24160

Pre-let-7-e UGAGGUAGGAGGUUGUAUAGUUGAGGAGGACACCCAAGGAGAUCACUAUACGGCCUCCUAGCUUUCC

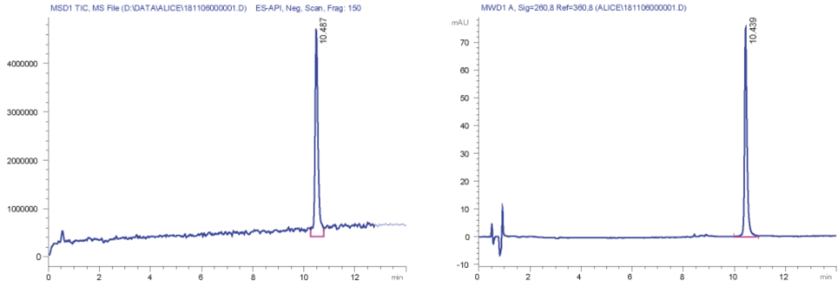

Deconvolution of Spectrum # 1 @ 10.429 - 10.554 min

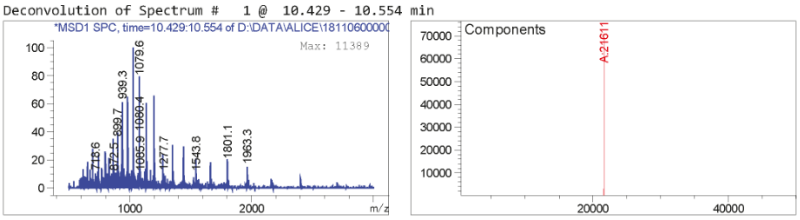

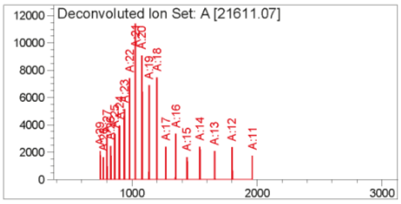

Component	Molecular Weight	Absolute Abundance	Relative Abundance
A	21611.07	68439	100.00

*** End of Report ***

Calculated MS: 21610

Pre-let-7-g GAGGUAGUAGUUUGUACAGUUUGAGGGUCUAUGAUACCACCCGGUACAGGAGAUAACUGUACAGGCCACUGCCUUGC

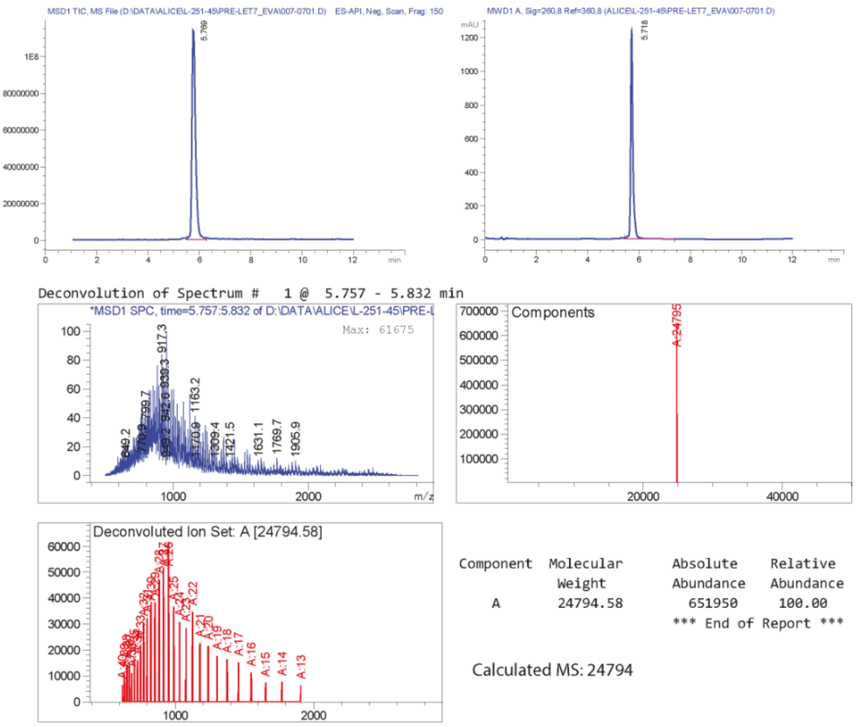

Supplementary Figure 28. LCMS chromatograms of oligonucleotides.

Reverse-phase high performance liquid chromatography mass spectrometry (LCMS) chromatograms of oligonucleotide sequences used in this study.

Supplementary Table 1

List of primers used in quantitative real-time PCR.

Mouse	Forward 5' ⟶ 3'	Reverse 5' ⟶ 3'
PPARα	ACCACTACGGAGTTCACGCATG	GAATCTTGCAGCTCCGATCACAC
Hmgcs2	TGCTATGCAGCCTACCGCAAGA	GCCAGGGATTTCTGGACCATCT
Hmgcl	GCACTTTGCCAAAGCAGGTGAAG	CGGAAAGCATGTCGATCAGCCT
Bdh1	AGGCTGTGACTCTGGATTTGGG	CTGGATGGTTCTCAGTCGGTCA
Cpt1a	GGCATAAACGCAGAGCATTCCTG	CAGTGTCCATCCTCTGAGTAGC
Fgf21	ATCAGGGAGGATGGAACAGTGG	AGCTCCATCTGGCTGTTGGCAA
Srebp1c	GGA GCC ATG GAT TGC ACA TT	GGC CCG GGA AGT CAC TGT
Acc	TGA CAG ACT GAT CGC AGA GAA AG	TGG AGA GCC CCA CAC ACA
Acs	CGC ACC CTT CCA ACC AAC A	CGC TAT TTC CAC TGA CTG CAT
Acl	ACT TTC TCA TTG AAC CCT TCG TC	TCC ACA TCG CCC ACA TCC AC
Scd1	CCG GAG ACC CCT TAG ATC GA	TAG CCT GTA AAG ATT TCT GCA AAC C
TNFa	CCA AGG CGC CAC ATC TCC CT	GCT TTC TGT GCT CAT GGT GT
IL1b	CCA AGC AAC GAC AAA ATA CC	GTT GAA GAC AAA CCG TTT TTC C
Ccl2	AGC AAG ATG ATC CCA ATG AGT	GAG CTT GGT GAC AAA AAC TAC AG
Cxcl10	CCC ACG TGT TGA GAT CAT TG	CAG TTA AGG AGC CCT TTT AGA CC
Col1a1	ACA TGT TCA GCT TTG TGG ACC	TAG GCC ATT GTG TAT GCA GC
Timp1	CCC TGC TCA GCA AAG AGC	TCA CTC TCC AGT TTG CAA GG
TGFb	TCA CTC TCC AGT TTG CAA GG	GCC TTA GTT TGG ACA GGA TCT G
β-Actin	CATTGCTGACAGGATGCAGAAGG	TGCTGGAAGGTGGACAGTGAGG
Human		

PPARα	CTC GTG AAT TCC TAA AAA GCC TA	TTT AGA AGG CCA GGA CGA TCT
Cpt1a	CAT GTC CAG CCA GAC GAA GA	TGG TGG CAC GAC TCA TCT TG
Mcad	GAG CAG GCT CTG ATG TAG CTG G	GTA TCT GAA CAT CGC TGG CCC
Hmgcl	TCC ACT GCC ATG ACA CCT ATG	AAG CCC TCT AGC ATG TAG ACC
Bdh1	GGC AGA AGT GAA CCT TTG GG	GCA GTC CGA GAA AGC CTC TAC
Acc	GCC GCT TGC CTG ACT TTT G	GTC TGG TTC ATC CAC GAG CA
Acly	CGG ACT TCG GCA GAG GTA GA	GGA GTT CTT TGC CCG TCT GC
Fasn	AAG GAC CTG TCT AGG TTT GAT GC	TGG CTT CAT AGG TGA CTT CCA
Scd1	AGG CAG CCT CCT TTG TGT GT	AGG GTT TGC CAG CCT TGT CT
NCoR1	GGA GCA GAA ACA CCG CAG TA	TCA CCT GGT TTG TCT TGA TGT
β-Actin	AGC GAG CAT CCC CCA AAG TT	GGG CAC GAA GGC TCA TCA TT
