# Supplementary material for: Pharmacological inhibition of Lin28 promotes ketogenesis and restores lipid homeostasis in models of non-alcoholic fatty liver disease
Source: Nat Commun. 2022 Dec 26;13:7940. doi: 10.1038/s41467-022-35481-1 (PMC9792516; doi:10.1038/s41467-022-35481-1)
Supplement: Supplementary file 2 — Reporting Summary [file 41467_2022_35481_MOESM2_ESM.pdf]

nature portfolio

Corresponding author(s): Carlo V. Catapano and Jonathan Hall

Last updated by author(s): Jun 15, 2022

Reporting Summary

Nature Portfolio wishes to improve the reproducibility of the work that we publish. This form provides structure for consistency and transparency in reporting. For further information on Nature Portfolio policies, see our Editorial Policies and the Editorial Policy Checklist.

Statistics

For all statistical analyses, confirm that the following items are present in the figure legend, table legend, main text, or Methods section.

n/a	Confirmed	
☐	☒	The exact sample size (*n*) for each experimental group/condition, given as a discrete number and unit of measurement
☐	☒	A statement on whether measurements were taken from distinct samples or whether the same sample was measured repeatedly
☐	☒	The statistical test(s) used AND whether they are one- or two-sided<br>*Only common tests should be described solely by name; describe more complex techniques in the Methods section.*
☒	☐	A description of all covariates tested
☐	☒	A description of any assumptions or corrections, such as tests of normality and adjustment for multiple comparisons
☐	☒	A full description of the statistical parameters including central tendency (e.g. means) or other basic estimates (e.g. regression coefficient) AND variation (e.g. standard deviation) or associated estimates of uncertainty (e.g. confidence intervals)
☐	☒	For null hypothesis testing, the test statistic (e.g. F, t, r) with confidence intervals, effect sizes, degrees of freedom and P value noted<br>*Give P values as exact values whenever suitable.*
☒	☐	For Bayesian analysis, information on the choice of priors and Markov chain Monte Carlo settings
☒	☐	For hierarchical and complex designs, identification of the appropriate level for tests and full reporting of outcomes
☒	☐	Estimates of effect sizes (e.g. Cohen's *d*, Pearson's *r*), indicating how they were calculated

Our web collection on statistics for biologists contains articles on many of the points above.

Software and code

Policy information about availability of computer code

Data collection	Leica DMI6000 Confocal Laser Scanning Microscope System was used in the Oil Red O Staining assay. The histology images were acquired via Leica Aperio AT2.A Flow cytometer (BD LSR Fortessa) was used for the 2-NBDG Glucose Uptake Assay. A ChemiDoc™ XRS Imaging System (Biorad) was used for chemiluminescence imaging. A SpectraMax Paradigm plate reader (Molecular Devices) was used for spectrophotometric assays. Targetscan software was used to examine potential target sites of let-7 in NCoR1 mRNA. A LightCycler 480 Real-Time PCR system (Roche) was used for RT-PCR and Taqman PCR assays.
Data analysis	Graphpad Prism (version 7.0) was used for graphing data and statistical analysis, Image J (version 1.52p) was used for analyzing western blot images and histology pictures. FlowJo (version 10) was used for Flow Cytometry analysis.

For manuscripts utilizing custom algorithms or software that are central to the research but not yet described in published literature, software must be made available to editors and reviewers. We strongly encourage code deposition in a community repository (e.g. GitHub). See the Nature Portfolio guidelines for submitting code & software for further information.

Data

Policy information about availability of data

All manuscripts must include a data availability statement. This statement should provide the following information, where applicable:

- Accession codes, unique identifiers, or web links for publicly available datasets
- A description of any restrictions on data availability
- For clinical datasets or third party data, please ensure that the statement adheres to our policy

Data are available within the Article or Supplementary Information. Source data are provided with this paper.

Human research participants

Policy information about studies involving human research participants and Sex and Gender in Research.

Reporting on sex and gender	N/A
Population characteristics	N/A
Recruitment	N/A
Ethics oversight	N/A

Note that full information on the approval of the study protocol must also be provided in the manuscript.

Field-specific reporting

Please select the one below that is the best fit for your research. If you are not sure, read the appropriate sections before making your selection.

☒ Life sciences ☐ Behavioural & social sciences ☐ Ecological, evolutionary & environmental sciences

For a reference copy of the document with all sections, see nature.com/documents/nr-reporting-summary-flat.pdf

Life sciences study design

All studies must disclose on these points even when the disclosure is negative.

Sample size	Included in Figure legends. Sample sizes were determined based on our prior experience with similar in vitro and in vivo studies. The number of mice used was sufficient for acceptable statistical analyses, without sacrificing too many animals. Experiments were performed with at least 3 independent replicates.
Data exclusions	A serum sample contaminated with blood was excluded from Fig.2a.
Replication	Indicated in figure legends
Randomization	Mice were randomly divided into different treatment groups. Cells were grown under the same conditions and randomly allocated into different treatment groups without any bias.
Blinding	In this study blinding was not possible because treatment of animals/cells and biochemical analyses were frequently done by the same investigators

Reporting for specific materials, systems and methods

We require information from authors about some types of materials, experimental systems and methods used in many studies. Here, indicate whether each material, system or method listed is relevant to your study. If you are not sure if a list item applies to your research, read the appropriate section before selecting a response.

Materials & experimental systems

n/a	Involved in the study
☐	☒ Antibodies
☐	☒ Eukaryotic cell lines
☒	☐ Palaeontology and archaeology
☐	☒ Animals and other organisms
☒	☐ Clinical data
☒	☐ Dual use research of concern

Methods

n/a	Involved in the study
☒	☐ ChIP-seq
☐	☒ Flow cytometry
☒	☐ MRI-based neuroimaging

Antibodies

Antibodies used

Anti-Lin28A (clone D9F5) rabbit monoclonal antibody, Cell Signaling, Cat # 8706, Western Blot (WB) (1:1000)
Anti-Lin28b rabbit polyclonal antibody, Cell Signaling, cat # 4196, WB (1:1000)
Anti-Lin28b (mouse preferred) rabbit polyclonal antibody, Cell Signaling, Cat # 5422, WB (1:1000)
Anti-Phospho-Insulin Receptor β (Tyr1361) (clone 84B2) rabbit monoclonal antibody, Cell Signaling, Cat #3023, WB (1:1000)
Anti-insulin receptor beta mouse monoclonal antibody (clone CT-3), Santa Cruz, Cat # sc-57342, WB (1:1000)
Anti-total-Akt (C67E7) rabbit monoclonal antibody, Cell Signaling, Cat # 4691, WB (1:1000)
Anti-phospho-Akt Ser473 (clone D9E) rabbit monoclonal antibody, Cell Signaling, Cat # 4060, WB (1: 1000)
Anti-α-Tubulin (clone B-7) mouse monoclonal antibody, Santa Cruz, Cat # sc-5286, WB (1:1000)
Anti-NCoR1 rabbit polyclonal antibody, Proteintech, Cat # 20018-1-AP, WB (1:1000)
Anti-NCoR1 rabbit polyclonal antibody, Cell Signaling, Cat # 5948, WB (1:1000)
Anti-β-Actin (clone C4) mouse monoclonal antibody, Santa Cruz, sc-47778, WB (1: 1000)
Anti-Pten (clone A2B1) mouse monoclonal antibody, Santa-Cruz, Cat # sc-7974, WB (1:1000)
Anti-PPARα (clone 1G1E10) mouse monoclonal antibody, Proteintech, Cat # 66826-1-Ig, WB (1: 2000)
Anti-GAPDH (clone 1E6D9) mouse monoclonal antibody, Proteintech, Cat # 60004-1-Ig, WB (1:50000)
Anti-SREBP1 (clone 1B6G5) mouse monoclonal antibody, Proteintech, Cat # 66875-1-Ig, WB (1:2000)
Anti-Phospho-RPS6 (Ser235) (clone 2A4B6) mouse monoclonal antibody, Proteintech, Cat # 67898-1-Ig, WB (1:5000)
Anti-S6K2 rabbit polyclonal antibody, Proteintech, cat # 14823-1-AP, WB (1:1000)
Anti-Phospho-p44/42 MAPK (Erk1/2) (Thr202/Tyr204) (clone 197G2) rabbit monoclonal antibody, Cell Signaling, Cat #4377, WB, (1:1000)
Anti-p44/42 MAPK (Erk1/2) rabbit polyclonal antibody, Cell Signaling, Cat # 9102, WB (1:1000).
Anti-Perilipin 2 (N-terminus aa 1-16) guinea pig polyclonal antibody, Progen, Cat. # GP46, IP (1: 800)

Validation

Anti-Lin28A (Cell Signaling, 8706) was validated by Western Blot (WB) analyses of extracts from NTERA-2 cl.D1, NCCIT, F9 cells, and Immunofluorescence analysis (IF) of NTERA-2 cl.D1 and HeLa cells. The antibody was also validated in our lab by WB in Lin28a-overexpressing cells.
Anti-Lin28b (Cell Signaling, 4196) was validated by Western blot analysis of extracts from HepG2 and NTERA-2 cells, and human testes. We validated this antibody by WB in HepG2 cells, untreated or treated with siRNA against Lin28b.
Anti-Lin28b (mouse preferred) was validated by WB in extracts from P19 and MES cells. We have validated this antibody in C2C12 cells, untreated or treated with siRNA against Lin28b.
Anti-Phospho-Insulin Receptor β (Tyr1361) (84B2) (Cell Signaling, 3023) was validated by Western blot analysis of CHO cells overexpressing human insulin receptors untreated or treated with insulin.
Anti-insulin receptor beta (Santa Cruz, sc-57342) was validated by WB analysis in NIH/3T3, SW480, MCF7, JAR, MIA PaCa-2 and HepG2 whole cell lysates.
Anti-total-Akt (Cell Signaling, 4691) was validated by WB from extracts of various cell lines, by Immunohistochemical analysis of paraffin-embedded human melanoma, human breast carcinoma, by Confocal immunofluorescent analysis of C2C12 cells, LY294002-treated or insulin-treated, by Flow cytometric analysis of Jurkat cells.
Anti-phospho-Akt Ser473 (Cell Signaling, 4060) was validated by WB in extracts from PC-3 cells, untreated or LY294002/wortmannin-treated, and NIH/3T3 cells, serum-starved or PDGF-treated. It has been validated by Immunoprecipitation in Jurkat extracts treated with Calyculin. It has been validated by immunohistochemical analysis of paraffin-embedded PTEN heterozygous mutant mouse endometrium. It has been validated by confocal immunofluorescent analysis of C2C12 cells, LY294002-treated or insulin-treated. It has been validated by Flow cytometric analysis of Jurkat cells, untreated or treated with LY294002, Wortmannin, and U0126. We have finally validated it by Western Blot analysis of HepG2 cells, with or without insulin stimulation.
Anti-α-Tubulin (B-7), (Santa Cruz, sc-5286) was validated by Western blot analysis in K-562, HEL92.1.7, RAW 264.7, C2C12, PC-12 and A-10 whole cell lysates and Immunofluorescence staining of HeLa cells.
Anti-NCoR1 (Proteintech, 20018-1-AP) was validated by WB in HepG2, and K-562 cells.
Anti-NCoR1 (Cell Signaling, 5948) was validated by WB in extracts from Jurkat, U-251, U-87 MG, SW 480, C2C12, Neuro-2a, PC12,

COS-7 cells.
Anti-β-Actin (Cell Signaling, sc-47778) was validated by Fluorescent western blot analysis in Jurkat, HeLa, and A-431 whole cell lysates and by Immunoperoxidase detection in formalin-fixed, paraffin-embedded human smooth muscle tissue, and human tonsil tissue.
Anti-Pten (A2B1) (Santa-Cruz, sc-7974) was validated by Western blot analysis in SH-SY5Y, MCF7, and C3H/10T1/2 whole cell lysates and mouse brain tissue extract. It was also analysed by Immunofluorescence staining in HeLa cells. We have validated this antibody in PTEN-deficient iver extracts.
Anti-PPARa, (Proteintech, 66826-1-Ig) was validated by WB in extracts from HepG2, and HeLa cells.
Anti-GAPDH (Proteintech, 60004-1-Ig) was validated by WB in HeLa, HepG2, ROS1728, pig brain tissue, zebrafish tissue, whole yeast, whole Nematode tissue, soybean whole plant tissue, arabidopsis whole plant tissue, HEK-293, Jurkat, K-562, HSC-T6, NIH/3T3, 4T1, C6, PC-12, C2C12, SP2/0 cells, rat brain tissue, mouse brain tissue, by Immunoprecipitation in HeLa cells, by Immunofluorescence in Ethacrynic acid treated HeLa cells and Flow Cytometry in HeLa cells.
Anti-SREBP1 (Proteintech, 66875-1-Ig) was KD/KO validated. It was also validated by Western Blot (WB) in A549, HeLa, Jurkat, L02, and HT-29 cells, by Immunohistochemistry (IHC) in human kidney tissue and by Immunofluorescence (IF) in HepG2 cells.
Anti-Phospho-RPS6 (Ser235) (Proteintech, 67898-1-Ig) was validated by WB in extracts from HeLa, HEK-293, Calyculin A treated HeLa cells, Calyculin A treated HEK-293 cells, IGF-1 treated MCF-7 cells, Calyculin A treated NIH/3T3 cells.
Anti-S6K2 was validated by WB in MCF7 cells, by Immunoprecipitation (IP) in MCF-7 cells, by Immunohistochemistry (IHC) in human kidney tissues, and by Immunofluorescence (IF) in MCF-7 cells.
Anti-Phospho-p44/42 MAPK (Erk1/2) (Thr202/Tyr204) was validated by WB in extracts from NIH/3T3 cells treated with UV light and PDGF, by Confocal immunofluorescent analysis of HT-1080 cells, starved overnight then treated with U0126 or PDBu (Phorbol 12,13-Dibutyrate) and by Flow cytometric analysis of Jurkat cells, treated with U0126 or treated with TPA (12-O- Tetradecanoylphorbol-13 -Acetate).
Anti-p44/42 MAPK (Erk1/2) (Cell Signaling, 9102) was validated by Western blot analysis of extracts from serum-induced PC12 cells and by Immunohistochemical analysis of paraffin-embedded human breast carcinoma.
Anti-Perilipin 2 (Progen, GP46) was validated by Western blot analysis of undifferentiated 3T3-L1 and differentiated 3T3-L1 ALC (= adipocyte like cells), and HepG2 cell lysates, by Immunofluorescence analysis of HepG2 cells.

Eukaryotic cell lines

Policy information about cell lines and Sex and Gender in Research

Cell line source(s)

Human HepG2 (ATCC, HB-8065), AML12 (ATCC, CRL-2254), HEK293T (ATCC, CRL-3216) and mouse C2C12 (ECACC 91031101) cell lines. Cell lines were obtained from American Type Culture Collection (ATCC).

Authentication

Commercial HepG2 and HEK293T cells were authenticated by STR profiling/ ATCC

Mycoplasma contamination

Cell lines were tested negative for Mycoplasma contamination.

Commonly misidentified lines
(See ICLAC register)

No commonly misidentified cell lines were used for this study.

Animals and other research organisms

Policy information about studies involving animals; ARRIVE guidelines recommended for reporting animal research, and Sex and Gender in Research

Laboratory animals

Study protocols were approved by the Swiss Veterinary Authority (license number:31966).
Mice were obtained by Charles River Laboratories (C57BL/6J; Crl:NU(NCr)-Foxn1nu) or generated in house (Alb-Cre;Ptenflox/flox).
The mice were maintained in pathogen-free conditions on a 12h light/dark cycle at 20-24°C and 50-65% relative humidity.
7-week old C57BL/6J wild type mice were treated with C1632 (50mg/kg) or vehicle for 5 consecutive days
7-week old nude mice were treated with C1632 (50mg/kg) or vehicle for 5 consecutive days
7-week old nude mice were treated with C1632 (50mg/kg) or vehicle for 3 weeks (3 IP injections per week)
6-week-old Alb-Cre;Ptenflox/flox mice were treated for 4 weeks with vehicle or 1632 and they were sacrificed at the end of the treatment.
22 week-old Alb-Cre;Ptenflox/flox mice were treated with vehicle or 1632 for 6 weeks and they were sacrificed at the end of the treatment.
6-week-old C57BL/6J mice were fed with a Rodent Diet with 40 kcal% fat (mostly palm oil), 20 kcal% fructose and 2% cholesterol supplied from ResearchDiet (#D09100310) for 11 weeks. During the last 4 weeks of the NAFLD diet mice were randomly assigned to receive vehicle or C1632 and they were sacrificed at the end of the treatment.

Wild animals	The study did not include wild animals
Reporting on sex	All the mice used for this study were male as described in the Methods
Field-collected samples	No field-collected samples were used in the study.
Ethics oversight	Study protocols were approved by the Swiss Veterinary Authority (license number:31966).

Note that full information on the approval of the study protocol must also be provided in the manuscript.

Flow Cytometry

Plots

Confirm that:

☒ The axis labels state the marker and fluorochrome used (e.g. CD4-FITC).

☒ The axis scales are clearly visible. Include numbers along axes only for bottom left plot of group (a 'group' is an analysis of identical markers).

☒ All plots are contour plots with outliers or pseudocolor plots.

☒ A numerical value for number of cells or percentage (with statistics) is provided.

Methodology

Sample preparation	HepG2 cells were seeded in six-well plates (8×105 cells/well) in DMEM/F-12 GlutaMAXTM (Gibco) supplemented with 10% of FBS and 1% antibiotics (Penicillin/Streptomycin). Cells were treated with 100 µM of C1632 for 4 days. On the day of the flow cytometry assay cells were incubated in glucose-free DMEM medium (Gibco) at 37° C for 2 h. Cells were then incubated with 200µM of 2-[N-(7-nitrobenz-2-oxa-1,3-diazol-4-yl)amino]-2-deoxy-D-glucose (2-NBDG) (Biovision) at 37° C for 45min. Cells were washed with phosphate-buffered saline (PBS) and subsequently detached using trypsin for flow cytometry analysis. Cells were washed with 1 ml PBS prior to resuspension in 200 µl FACS buffer (2% FBS in PBS) and subsequently strained through CellTrics 50µm filters
Instrument	Flow Cytometer, CBD LSR Fortessa
Software	FlowJo
Cell population abundance	20000 single viable cells were measured for analysis every time
Gating strategy	Dead cells and debris, and cell doublets were excluded

☒ Tick this box to confirm that a figure exemplifying the gating strategy is provided in the Supplementary Information.
